# Systematic review and meta-analysis of insecticide resistance status and mechanisms in the arbovirus vector *Aedes aegypti* from Nigeria

**DOI:** 10.1371/journal.pntd.0014421

**Published:** 2026-06-15

**Authors:** Aisha Kabir Mohammed, Maryam Abdulkadir Dangambo, Aliyu Muhammad, Adamu Jibrin Alhassan

**Affiliations:** 1 Department of Biochemistry, Faculty of Basic Medical Sciences, Bayero University Kano, Kano, Kano State, Nigeria; 2 Department of Biochemistry, Faculty of Life Sciences, Ahmadu Bello University Zaria, Zaria, Kaduna State, Nigeria; Institut de recherche pour le developpement, FRANCE

## Abstract

**Background:**

Arboviruses including dengue, Zika, and chikungunya pose a growing health threat in Nigeria, where *Aedes aegypti* is the main vector. Insecticide-based control is increasingly undermined by resistance. Although individual studies have reported resistance, no systematic review has synthesized these findings. This study assessed the prevalence, distribution, and mechanisms of insecticide resistance in Nigerian *Aedes* populations.

**Methodology:**

We conducted a systematic review and meta-analysis following PRISMA 2020 guidelines, with protocol registered on the Open Science Framework (https://doi.org/10.17605/OSF.IO/CTUH8). Searches across PubMed, Scopus, Web of Science, Google Scholar, AJOL, and VectorBase identified studies from Nigeria. Eligible studies examined field-collected *Aedes aegypti* populations for insecticide susceptibility via WHO bioassays or resistance mechanisms such as kdr mutations and metabolic enzyme activity. Data were pooled using random-effects models, with heterogeneity assessed by I² and Cochran’s Q. Nine studies published between 2015 and 2025 met inclusion criteria.

**Results:**

Pooled estimates showed entrenched resistance to pyrethroids (75.6%, 95% CI: 40.5–93.4) and DDT (28.0%, 95% CI: 6.6–68.3), consistently below WHO thresholds. Carbamates displayed variable susceptibility (91.1%, 95% CI: 22.6–99.7), ranging from full susceptibility in Abia to resistance in Kogi. Organophosphates had the highest pooled mortality (98.3%, 95% CI: 96.8–99.1), though emerging resistance was reported in several states. Mechanistic evidence implicated frequent F1534C kdr mutations and elevated detoxification enzyme activity.

**Conclusion:**

This review confirms widespread insecticide resistance in *Ae. aegypti* across Nigeria, especially to pyrethroids and DDT, with regional variation in carbamate susceptibility and emerging organophosphate resistance. The findings emphasize the urgent need for adaptive vector control strategies, expanded surveillance in northern regions, and integration of genomic tools to better characterize resistance mechanisms. Nigeria’s resistance profile mirrors challenges faced in many low- and middle-income countries, reinforcing the global imperative to strengthen monitoring systems and sustain effective arbovirus vector control.

## Author summary

Mosquitoes of the *Aedes* species, especially *Aedes aegypti*, spread viruses such as dengue, Zika, and chikungunya. In Nigeria, these diseases are becoming more common, but controlling the mosquitoes is increasingly difficult because they are resistant to many insecticides. We reviewed and combined evidence from nine studies conducted across different regions of Nigeria to understand how widespread this resistance is and what causes it. Our analysis showed that *Ae. aegypti* are highly resistant to pyrethroids and DDT, while resistance to carbamates and organophosphates varies by location. Genetic changes in the mosquitoes, known as kdr mutations, and increased activity of detoxifying enzymes both contribute to this resistance. These findings highlight that current control methods are losing effectiveness and that new strategies are urgently needed. Expanding mosquito surveillance, especially in northern Nigeria, and using modern genetic tools will help track resistance and guide better control programs. Nigeria’s experience reflects a wider global challenge in protecting communities from mosquito‑borne diseases.

## 1 Introduction

Arboviruses, including dengue, Zika, and chikungunya, constitute a major and persistent global public health crisis [1]. In Nigeria, the key vectors, *Aedes aegypti* and *Aedes albopictus*, thrive in urban and peri-urban environments, contributing to confirmed active dengue circulation and the co-circulation of multiple DENV serotypes across key urban centers such as Maiduguri [2], Ibadan [3], Abuja [4] and the densely populated Lagos metropolis [5,6]. This increasing disease incidence is intrinsically linked to rapid, often unplanned, urbanization and unpredictable climate variability [7–9].

Effective vector control relies heavily on insecticide-based interventions including indoor residual spraying, space spraying, and larval control measures; however, their efficacy is severely compromised by widespread insecticide resistance [6,10]. In Nigeria, integrated vector management (IVM) approaches are being piloted, including larval source management, environmental sanitation, and the use of synergist‑based formulations (e.g., pyrethroids combined with PBO) to partially restore susceptibility. Surveillance programs are expanding in some states, though coverage remains limited [11–13].

This resistance poses a significant threat to the sustainability of vector control programs for both arbovirus and malaria control [8,14]. Resistance to pyrethroids (e.g., permethrin and deltamethrin) is particularly prevalent, a phenomenon driven by intense selection pressure from public health and agricultural pesticide applications, which confirm that *Ae. aegypti* populations have established multiple resistance profiles to various insecticide classes, including organochlorines (DDT) and pyrethroids [15,16].

This resistance is primarily mediated by two key mechanisms: target-site insensitivity, known as knockdown resistance (kdr), which arises from mutations in the voltage-sensitive sodium channel gene. Specific kdr mutations, notably F1534C, S989P, and V1016I/L, have been confirmed in Nigerian *Ae. aegypti* populations [16–19].

Metabolic Resistance involves the overexpression of detoxifying enzymes. Research in Lagos State and some parts of Nigeria has specifically implicated the enhanced activity of Cytochrome P450 monooxygenases and Glutathione S-transferases as crucial contributing factors to the observed resistance phenotype against DDT and permethrin [16,18,20].

Given the rapidly evolving and geographically widespread nature of this resistance, a comprehensive and quantitative synthesis of current data is essential. Therefore, this systematic review and meta-analysis aim to quantitatively and qualitatively synthesize all available evidence on the insecticide resistance status and underlying molecular/metabolic mechanisms of *Ae. aegypti* across Nigeria to generate actionable evidence for national vector control policies.

## 2 Methods

### 2.1 Ethics statement

As this review utilizes publicly available data, ethical approval for each included study was verified. Studies were assessed for adherence to ethical guidelines for research involving human subjects. Any concerns regarding ethical standards were noted in the quality assessment phase.

### 2.2 Objectives and hypothesis

The objective of this review was to systematically assess the insecticide resistance status and underlying mechanisms in *Ae. aegypti* populations from Nigeria. We hypothesized that resistance prevalence varies across regions and insecticide classes, reflecting both geographic heterogeneity and differences in insecticide use patterns.

### 2.3 Reporting guidelines and protocol registration

This systematic review and meta-analysis was conducted and reported according to the guidelines of the Preferred Reporting Items for Systematic Reviews and Meta-Analyses (PRISMA) 2020 statement [21]. The full protocol for this review was registered *a priori* on the Open Science Framework (OSF) platform (https://doi.org/10.17605/OSF.IO/CTUH8).

### 2.4 Search strategy and data management

**Fig 1:**
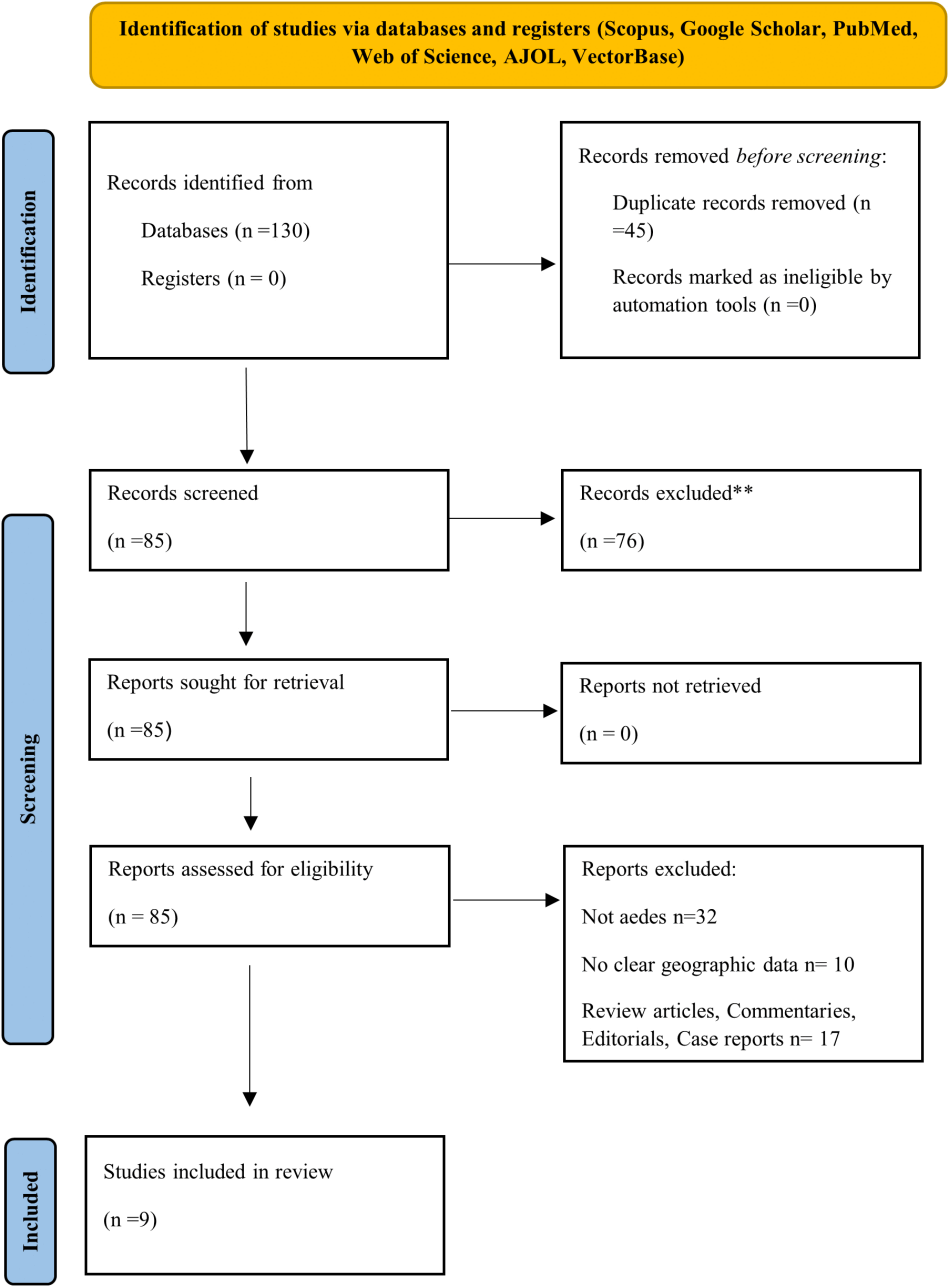
Prisma Flow Diagram.

A comprehensive literature search was performed across multiple electronic databases, including PubMed, Scopus, Google Scholar, Web of Science, African Journals Online (AJOL), and VectorBase. The search strategy employed key terms such as “*Aedes aegypti*,” “*Aedes albopictus*,” and “insecticide resistance,” utilizing Boolean operators (AND/OR) to optimize results. To ensure relevance, the search was restricted to publications from Nigeria. Further references were identified through citation tracking in key studies, ensuring comprehensive coverage of insecticide resistance and vector adaptation trends as shown in Fig 1.

All records were imported into Zotero reference management software, and duplicates were removed. The combined search yielded 130 records, resulting in 85 unique papers for screening.

Fig 1 summarizes the study selection process for the systematic review. A total of 130 records were identified from databases, with 45 duplicates removed. After screening 85 records, 76 were excluded for specific reasons: Not Aedes (n = 32), No clear geographic data (n = 10), and Review articles, Commentaries, Editorials, Case reports (n = 17). Finally, 9 studies met eligibility criteria and were included in the review.

### 2.5 Eligibility criteria

A modified PECO (Population, Exposure, Comparator, Outcome) framework was utilized to define the inclusion and exclusion criteria for relevant studies as seen in S2 File. We included studies on field-collected populations of *Ae. aegypti* from Nigeria that reported quantitative data on insecticide susceptibility status (24-hour mortality rates from WHO bioassays) or resistance mechanisms (*kdr* mutation frequencies or detoxifying enzyme activity levels). Studies on *Ae. albopictus* alone, laboratory-reared mosquitoes, or those lacking clear geographic data were excluded.

#### 2.5.1 Inclusion criteria.

The review included original research articles published in English that investigated insecticide resistance in *Aedes* mosquitoes primarily *Ae. aegypti* in Nigeria. Eligible studies reported resistance through bioassays (mortality rates from WHO protocols), genetic mechanisms (kdr mutation frequencies), or metabolic enzyme activity (mixed‑function oxidases and glutathione S‑transferases). The study population comprised field‑collected adult female *Ae. aegypti* mosquitoes from Nigeria, tested using standardized WHO bioassay procedures. This ensured that the review focused on natural mosquito populations directly relevant to arbovirus transmission risk.

#### 2.5.2 Exclusion criteria.

This review excluded studies that focused solely on non-*Aedes* mosquito species, as the objective was to assess insecticide resistance specific to *Aedes* in Nigeria. Review articles, commentaries, editorials, and case reports were not included to ensure that only primary research contributing original data was analyzed. Studies lacking sufficient methodological details or primary outcome data were excluded to maintain the integrity and reliability of the findings. Following title/abstract and full-text screening, 9 articles were included in the final synthesis.

### 2.6 Data extraction and risk of bias assessment

A standardized data extraction form was used to collect all relevant quantitative and qualitative data. The methodological quality and risk of bias for the nine included articles were assessed independently by two reviewers using the Joanna Briggs Institute (JBI) Critical Appraisal Tool for Prevalence Studies. All studies identified through the search were tabulated (S1 Table), with reasons for exclusion provided. Data extraction was performed using a standardized form (S2 Table). Risk of bias assessments are summarized in S3 Table.

#### 2.6.1 Risk of bias assessment.

The methodological quality and risk of bias for the nine included studies (N = 9) were assessed independently by two reviewers using the Joanna Briggs Institute (JBI) Critical Appraisal Tool for Prevalence Studies, chosen because the studies primarily focused on determining the prevalence (status) of insecticide resistance, an observational outcome. Disagreements were resolved by consensus.

### 2.7 Data synthesis and statistical analysis

The meta‑analysis was conducted in R using the metafor and meta packages. The primary effect size was defined as the pooled proportion of insecticide resistance, expressed through 24-hour mortality rates from WHO bioassays. To stabilize variances, proportions were transformed (logit or double arcsine) prior to pooling and subsequently back‑transformed for interpretation. A random‑effects model (restricted maximum likelihood [REML]) was applied to account for both within‑study error and between‑study heterogeneity. Heterogeneity was quantified using the I² statistic and Cochran’s Q test, with subgroup analyses stratified by insecticide class and geographic region. Publication bias was evaluated using funnel plots and Egger’s regression, with sensitivity analyses (including the Trim and Fill method) performed when bias was suspected. Resistance prevalence was quantified using WHO 24-hour bioassay mortality rates, which serve as internationally recognized screening‑level indicators of susceptibility. Although these assays do not yield resistance ratios relative to a susceptible reference strain, they remain the standard approach in systematic reviews and meta‑analyses for estimating prevalence patterns across mosquito populations. These statistical approaches are widely recommended for meta‑analyses of prevalence data, as they address variance instability, account for between‑study heterogeneity, and incorporate publication bias assessments, thereby ensuring robust and reliable pooled estimates.

## 3 Results

### 3.1 Study selection

The database search yielded 130 records. After removal of 45 duplicates, 85 unique studies were screened for eligibility. Following title/abstract and full‑text screening, 9 studies met the inclusion criteria and were retained for the final synthesis as seen in Fig 1 above. Details of all screened studies, including reasons for exclusion, are provided in S1 Table. Data extraction information, including extractor names, dates, and eligibility confirmation, is presented in S2 Table. Risk of bias assessments for the included studies, conducted using the JBI checklist for prevalence studies, are summarized in S3 Table.

### 3.2 Characteristics of included studies

This systematic review incorporated nine primary studies published between 2015 and 2025 that reported the insecticide susceptibility status of *Aedes aegypti* populations across Nigeria. The geographical coverage of these investigations predominantly spanned the southern and northern regions of the country, providing insight into the vector's status across six distinct states. The South-West region, specifically Lagos State, accounted for a substantial proportion of the published research. All included studies focused on the major arboviral vector, *Ae. aegypti*, although one regional assessment included other *Aedes* species (*Aedes spp.*) in its scope. Consistent testing across the collected populations involved the four major public health insecticide classes: Pyrethroids (e.g., Permethrin, Deltamethrin), Organochlorines (DDT), Carbamates (e.g., Bendiocarb), and Organophosphates (e.g., Primiphos-methyl). A summary of the key characteristics for the nine included studies is presented in Table 1.

**Table 1:** Characteristics of Included Studies on *Aedes aegypti* Insecticide Susceptibility in Nigeria (2015–2025).

Publication Year	Authors	Geographical Location (State)	Geo‑Political Zone	Target Species	Insecticide Classes Evaluated	Key Notes
**2015**	Ayorinde et al.	Lagos	South‑West	*Ae. aegypti*	Organochlorines, Pyrethroids	Early evidence of severe DDT and pyrethroid resistance.
**2018**	Ukpai & Ekedo	Umudike (Abia)	South‑East	*Ae. aegypti*	Organochlorines, Pyrethroids, Carbamates, Organophosphates	Susceptible to carbamates/organophosphates; resistant to DDT.
**2020**	Fagbohun, Idowu, Olakiigbe et al.	Lagos	South‑West	*Ae. aegypti*	Organochlorines, Pyrethroids, Carbamates	Variable carbamate susceptibility; strong pyrethroid/DDT resistance.
**2021**	Fagbohun et al.	Lagos	South‑West	*Ae. aegypti*	Pyrethroids (kdr mutations)	High frequency of F1534C mutation; rare S989P; co‑occurrence reported.
**2022**	Mukhtar & Ibrahim	Kano	North‑West	*Ae. aegypti*	Organochlorines, Pyrethroids, Carbamates, Organophosphates	Severe pyrethroid/DDT resistance; temephos resistance established.
**2024**	Ojianwuna et al.	Delta	South‑South	*Ae. aegypti*	Organochlorines, Pyrethroids	Resistance to lambda-cyhalothrin; PBO restored susceptibility.
**2024**	Sani Sade Muhammad et al.	Lokoja (Kogi)	North‑Central	*Ae. aegypti*	Organochlorines, Pyrethroids, Carbamates	Resistant to carbamates, pyrethroids, and DDT.
**2025**	Busari et al.	Osun	South‑West	*Ae. aegypti*	Organochlorines, Pyrethroids	Fully susceptible to all tested insecticides; no resistance detected.
**2025**	Nwangwu et al.	Ebonyi	South‑South / South‑East / South‑West	*Ae. aegypti* & spp.	Organochlorines, Pyrethroids, Carbamates, Organophosphates	Regional survey; confirmed widespread pyrethroid/DDT resistance; variable carbamate/organophosphate susceptibility.

**DDT** = Dichlorodiphenyltrichloroethane; **PBO** = Piperonyl butoxide (synergist that inhibits detoxifying enzymes); **F1534C** = Knockdown resistance mutation in the sodium channel gene (phenylalanine → cysteine at position 1534); **S989P** = Knockdown resistance mutation in the sodium channel gene (serine → proline at position 989).

### 3.3 Geographic distribution of included studies

**Fig 2:**
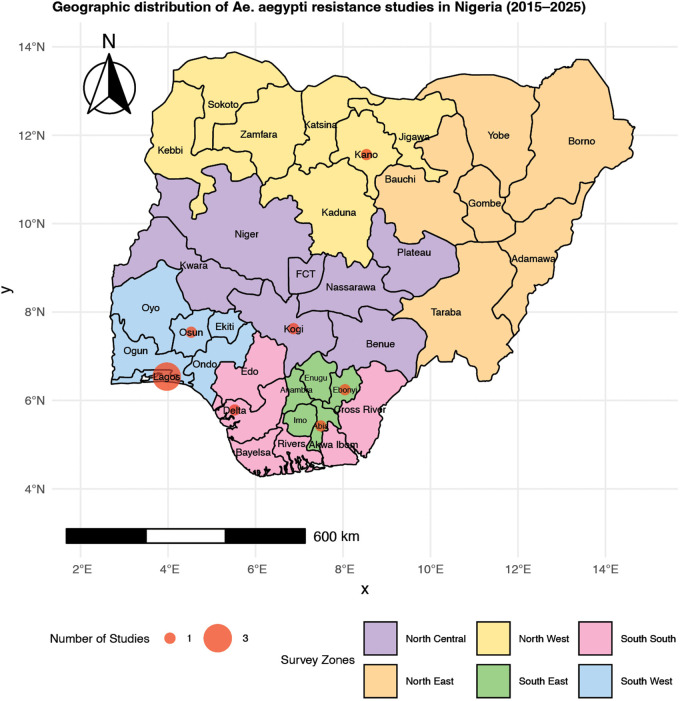
Ae. aegypti resistance studies in Nigeria, 2015–2025.

The map illustrates the geographic distribution of *Aedes aegypti* resistance studies conducted in Nigeria between 2015 and 2025. Different regions of the country are color‑coded by zone, while red circles mark the specific states where resistance studies have been carried out. The size of each circle reflects the number of studies performed, highlighting areas such as Lagos, Kano, Jigawa, Kogi, Enugu, and Cross River as key sites of research activity. This visualization underscores regional differences in study intensity and provides a spatial overview of mosquito resistance monitoring across Nigeria (Fig 2).

Geographic distribution of *Ae. aegypti* resistance studies in Nigeria (2015–2025). States are shaded by geopolitical zones: North Central (purple), North East (orange), North West (gold), South South (pink), South East (green), and South West (blue). Red circles indicate study locations, with circle size proportional to the number of studies. Base map shapefile: Natural Earth, public domain (http://www.naturalearthdata.com/about/terms-of-use/).

### 3.4 Insecticide susceptibility profile

Resistance status was assessed using WHO bioassay protocols, as detailed in the Methods section, with adult female mosquitoes tested for pyrethroids, organochlorines, carbamates, and organophosphates, and larval bioassays conducted for temephos. One study (Ojianwuna, 2024) additionally tested male *Ae. aegypti*, providing complementary data on resistance patterns.

#### 3.4.1 Pyrethroids.

Bioassays conducted across multiple Nigerian states consistently demonstrated high levels of resistance to pyrethroids, including permethrin, deltamethrin, and lambda-cyhalothrin. In Lagos, mortality rates for *Ae. aegypti* exposed to pyrethroids were frequently below the WHO susceptibility threshold of 90%, confirming resistance [22]. Similar findings were reported in Kano and Gombe, where resistance intensified between 2018 and 2020, with mortality rates declining over time [16]. Molecular analyses revealed the presence of knockdown resistance (kdr) mutations, particularly F1534C, which was detected at high frequency, and S989P, which was rare but significant. Importantly, the co‑occurrence of F1534C and S989P was documented for the first time in Africa [19]. Synergist assays using piperonyl butoxide (PBO) partially restored susceptibility, implicating cytochrome P450 monooxygenases in metabolic resistance [18].

#### 3.4.2 Organochlorines (DDT).

Resistance to DDT was extremely high across all surveyed regions. Mortality rates were consistently low, ranging from 20–30% in Lagos, Umudike (Abia), and Delta [23–25]. The northern Nigeria study confirmed similar patterns, with mortality well below WHO thresholds [16]. Mechanistic investigations linked DDT resistance to both kdr mutations and elevated glutathione S‑transferase activity, suggesting a combination of target-site insensitivity and metabolic detoxification [16].

#### 3.4.3 Carbamates (Bendiocarb).

Carbamate susceptibility varied geographically. In Umudike (Abia), *Ae. aegypti* populations remained fully susceptible, with mortality exceeding 98% [10]. In contrast, Lagos populations showed variable susceptibility, with mortality sometimes falling below 98%, indicating possible resistance [18]. In Lokoja (Kogi), resistance was pronounced, with mortality rates below 90% [26]. These findings highlight significant geographic heterogeneity in carbamate resistance across Nigeria.

#### 3.4.4 Organophosphates (Malathion, Pirimiphos‑methyl, Temephos).

Organophosphates generally performed better than pyrethroids and DDT, but resistance is emerging. In Lagos, malathion mortality rates were above 90%, yet moderate resistance was detected, with declining susceptibility over time [19]. Pirimiphos‑methyl resistance was observed at low levels in Ebonyi and Delta, where mortality rates fell below WHO thresholds [25,27]. Temephos resistance was established in Kano, with mortality dropping from ~50% in 2018 to ~34% in 2020, confirming a worsening trend [16]. These results suggest that while organophosphates remain more effective than pyrethroids and DDT, their reliability is diminishing in certain regions.

In Osun, *Ae. aegypti* populations were fully susceptible to all tested insecticides, including pyrethroids, carbamates, organophosphates, and DDT [28]. No evidence of resistance was detected, making Osun an outlier compared to other states where resistance is widespread.

#### 3.4.5 Regional trend.

In Lagos, *Ae. aegypti* populations exhibited high resistance to pyrethroids and DDT, with mortality rates consistently below WHO thresholds. The resistance mechanisms were largely due to target site mutations [22] and metabolic, as demonstrated by synergist assays with PBO, which partially restored susceptibility and confirmed the role of cytochrome P450 monooxygenases [18].

In Umudike, Abia State, mosquitoes remained fully susceptible to carbamates and organophosphates, with mortality rates exceeding 98%. However, resistance to DDT was evident, aligning with national trends of widespread organochlorine resistance [10].

In Delta State, resistance to lambda-cyhalothrin was detected, with mortality rates falling below WHO thresholds. Interestingly, PBO synergist assays restored susceptibility, highlighting the role of metabolic detoxification in pyrethroid resistance [25].

In Lokoja, Kogi State, *Ae. aegypti* populations were resistant to carbamates, pyrethroids, and DDT, with mortality consistently below 90%. This indicates a particularly challenging resistance profile in the region [26].

In Osun State, by contrast, mosquitoes were fully susceptible to all tested insecticides, including pyrethroids, carbamates, organophosphates, and DDT. No evidence of resistance was detected, making Osun an outlier compared to other states where resistance is widespread [28].

In Kano and Gombe, resistance to pyrethroids and DDT was severe and worsening over time. Between 2018 and 2020, mortality rates declined significantly, confirming that resistance is intensifying in northern Nigeria. Temephos resistance was also established, with mortality dropping below 50% by 2020 [16].

Finally, in Ebonyi State, overall resistance levels were low, but pirimiphos‑methyl resistance was emerging. Mortality rates fell below WHO thresholds, suggesting that organophosphate effectiveness may be diminishing in this region [27].

### 3.5 Forest plot of study-level mortality (%) stratified by insecticide class

**Fig 3:**
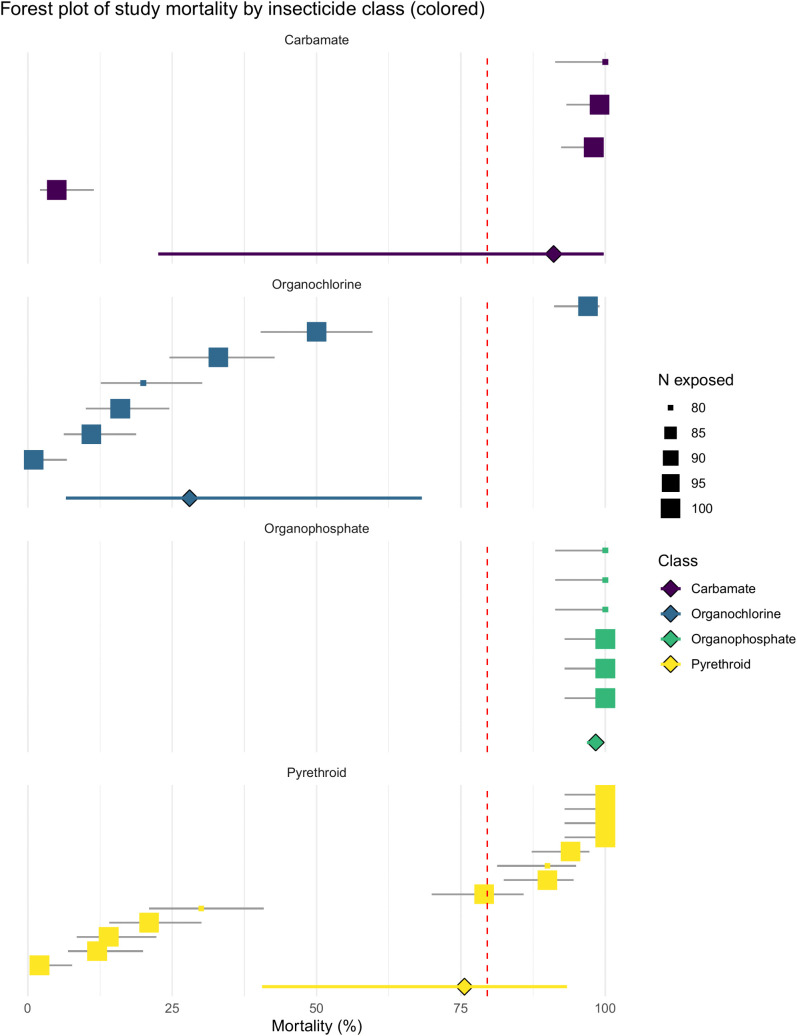
Forest plot of study-level mortality (%) stratified by insecticide class.

Fig 3 displays the forest plot of study-level mortality by insecticide class, with pooled proportional mortality (PPM) estimated using a random-effects REML model on logit-transformed proportions, subsequently back-transformed to percentages. Diamonds represent class-level pooled estimates with 95% confidence intervals. The pooled proportional mortality estimates revealed marked differences across insecticide classes, underscoring the heterogeneity of *Ae. aegypti* resistance in Nigeria. Pyrethroids demonstrated a pooled mortality of 75.6% (95% CI: 40.5–93.4; I² ≈ 98%), well below the WHO susceptibility threshold of 90%, confirming widespread resistance across Lagos, Kano, Delta, and Kogi. Organochlorines, represented by DDT, exhibited the lowest pooled mortality at 28.0% (95% CI: 6.6–68.3; I² ≈ 98%), reflecting severe resistance nationwide, with individual studies reporting mortality rates as low as 20–30% in Lagos, Abia, and Delta. Carbamates yielded a pooled mortality of 91.1% (95% CI: 22.6–99.7; I² ≈ 96%), but these aggregate masks strong geographic variation: full susceptibility in Umudike (Abia), possible resistance in Lagos (<98% mortality), and clear resistance in Lokoja (Kogi, < 90%). Organophosphates showed the highest pooled mortality at 98.3% (95% CI: 96.8–99.1; I² = 0%), suggesting overall effectiveness; however, individual studies revealed emerging resistance, including moderate malathion resistance in Lagos, low‑level pirimiphos‑methyl resistance in Ebonyi and Delta, and established temephos resistance in Kano, where mortality dropped below 50% by 2020. Taken together, the pooled estimates highlight a consistent pattern of severe resistance to pyrethroids and DDT, variable susceptibility to carbamates, and declining reliability of organophosphates despite their comparatively higher pooled mortality.

This forest plot presents comparative mortality outcomes for *Ae. aegypti* across four insecticide classes: carbamates, organochlorines, organophosphates, and pyrethroids. Each square denotes an individual study estimate, with square size proportional to the number of mosquitoes exposed (range: 80–100). Horizontal lines represent the 95% confidence intervals for each study. Class‑specific pooled summary estimates are illustrated by colored diamonds, enabling direct comparison of overall efficacy across insecticide categories. A vertical dashed red line marks the 75% mortality threshold, a benchmark commonly applied to assess insecticide effectiveness. Color coding facilitates interpretation: carbamates (purple), organochlorines (blue), organophosphates (green), and pyrethroids (yellow).

### 3.6 Pooled mortality estimates by insecticide class

Table 2 summarizes the pooled mortality estimates for *Ae. aegypti* across insecticide classes in Nigeria. Organochlorines (DDT) showed the lowest pooled mortality (28.0%, 95% CI: 6.6–68.3) with very high heterogeneity, confirming severe resistance nationwide. Pyrethroids also demonstrated entrenched resistance, with pooled mortality of 75.6% (95% CI: 40.5–93.4). Carbamates yielded a pooled mortality of 91.1% (95% CI: 22.6–99.7), but with substantial heterogeneity reflecting geographic variability. Organophosphates had the highest pooled mortality (98.3%, 95% CI: 96.8–99.1) and low heterogeneity, indicating overall effectiveness, though regional data revealed emerging resistance. The overall pooled mortality across all classes was 79.6% (95% CI: 57.5–91.8), underscoring widespread resistance and the urgent need for adaptive vector control strategies.

**Table 2:** Pooled Mortality Estimates by Insecticide Class (Nigeria, 2015–2025).

Insecticide Class	Pooled Mortality (%)	95% CI	References (Studies Contributing)	I² (%)	τ² (Between‑Study Variance)	Key Interpretation
Organochlorines (DDT)	28.0	6.6 – 68.3	Ayorinde 2015; Ukpai & Ekedo 2018; Fagbohun 2020; Mukhtar & Ibrahim 2022; Ojianwuna 2024; Sani Sade Muhammad 2024; Nwangwu 2025	≈ 98	High	Severe resistance nationwide; mortality often 20–30%.
Pyrethroids	75.6	40.5 – 93.4	Ayorinde 2015; Ukpai & Ekedo 2018; Fagbohun 2020; Fagbohun 2021; Mukhtar & Ibrahim 2022; Ojianwuna 2024; Sani Sade Muhammad 2024; Busari 2025; Nwangwu 2025 (13 datasets across these studies)	≈ 98	High	Entrenched resistance; mortality consistently <90%.
Carbamates (Bendiocarb)	91.1	22.6 – 99.7	Ukpai & Ekedo 2018; Fagbohun 2020; Sani Sade Muhammad 2024; Nwangwu 2025	≈ 96	High	Geographic heterogeneity: Abia susceptible, Lagos borderline, Kogi resistant.
Organophosphates (Malathion, Pirimiphos‑methyl, Temephos)	98.3	96.8 – 99.1	Ukpai & Ekedo 2018; Mukhtar & Ibrahim 2022; Ojianwuna 2024; Nwangwu 2025 (6 datasets across these studies)	0	Low	Overall effective, but resistance emerging (Lagos, Ebonyi, Delta, Kano).
Overall (All Classes)	79.6	57.5 – 91.8	All nine included studies (2015–2025)	High	High	Confirms widespread resistance, strongest impact on pyrethroids and DDT.

### 3.7 Predicted mortality over time by insecticide class

**Fig 4:**
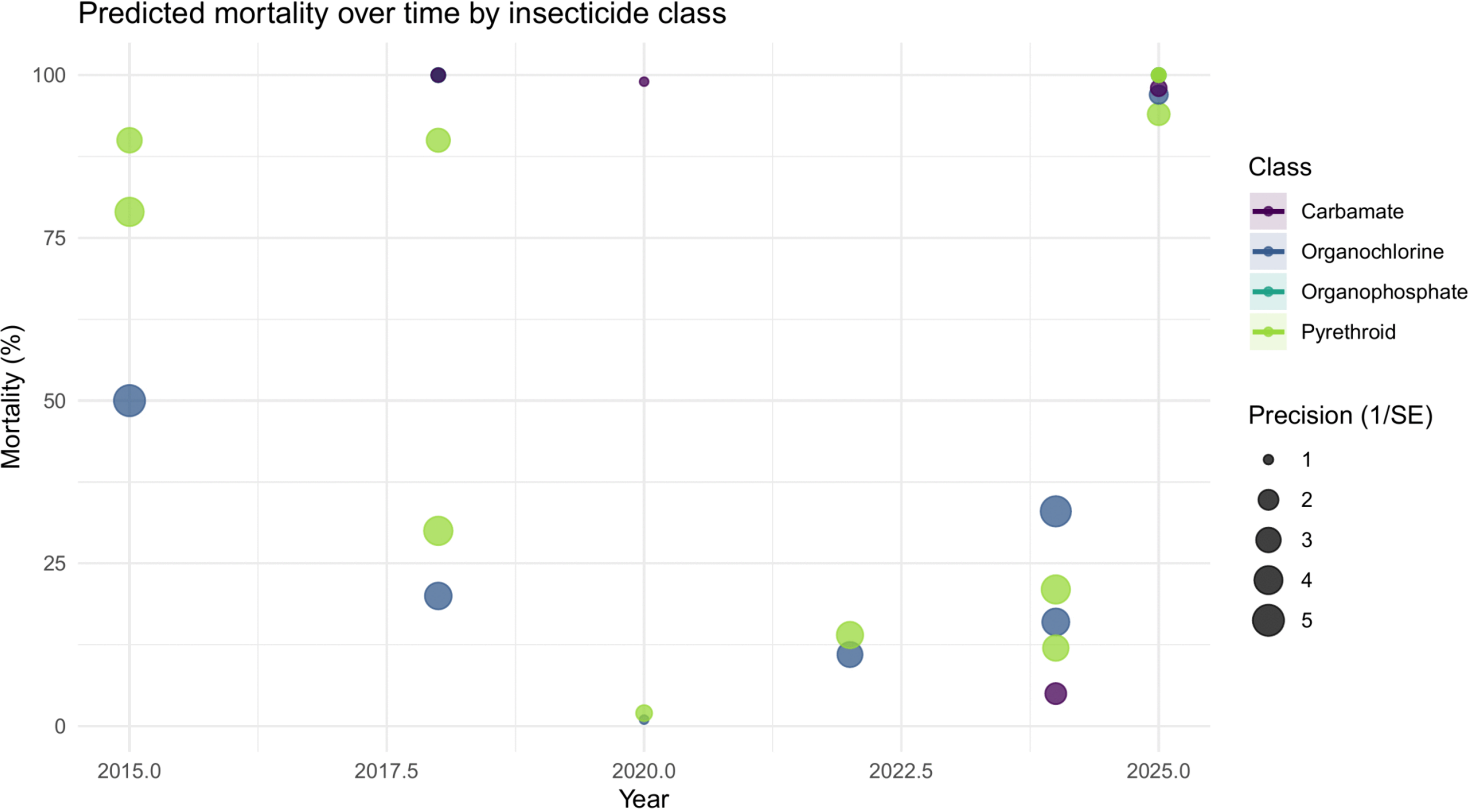
Predicted mortality over time by insecticide class.

Fig 4 illustrates predicted mortality trends over time by insecticide class, derived from a meta‑regression model fitted on the logit scale and back‑transformed to percentages. Model predictions are shown as lines with shaded ribbons representing 95% confidence intervals, while observed study points are plotted as bubbles scaled by precision (1/SE). Temporal meta‑regression revealed important trends in insecticide susceptibility across Nigerian *Ae. aegypti* populations. For pyrethroids, predicted mortality in 2025 was 80.6% (95% CI: 44.1–95.6), confirming entrenched resistance and suggesting that efficacy will remain below WHO thresholds. This aligns with longitudinal data from Kano and Gombe, where mortality rates steadily declined between 2018 and 2020, reflecting an intensifying resistance profile. For organochlorines, predicted mortality remained extremely low, consistent with the pooled estimate of 28%, underscoring the persistence of severe DDT resistance nationwide. Carbamates showed variable trends, with susceptibility maintained in Umudike (Abia) but declining in Lagos and clear resistance in Kogi, highlighting strong geographic heterogeneity. Organophosphates initially appeared highly effective, with pooled mortality near 98%, yet temporal data revealed emerging resistance: malathion susceptibility in Lagos declined over time, pirimiphos‑methyl resistance was detected in Ebonyi and Delta, and temephos resistance was firmly established in Kano, where mortality dropped from ~50% in 2018 to ~34% in 2020. These temporal patterns emphasize that while organophosphates currently outperform pyrethroids and DDT, their reliability is eroding, and resistance is spreading across regions.

Bubble plot showing predicted mortality percentages for *Ae. aegypti* from 2015 to 2025 across four insecticide classes: carbamates (purple), organochlorines (blue), organophosphates (teal), and pyrethroids (light green). Each point represents a study prediction, with circle size proportional to precision (1/SE). Larger circles indicate higher precision. The plot illustrates temporal variation in predicted mortality, highlighting differences in efficacy and potential resistance trends among insecticide classes.

### 3.8 Overall pooled proportional mortality (REML)

**Fig 5:**
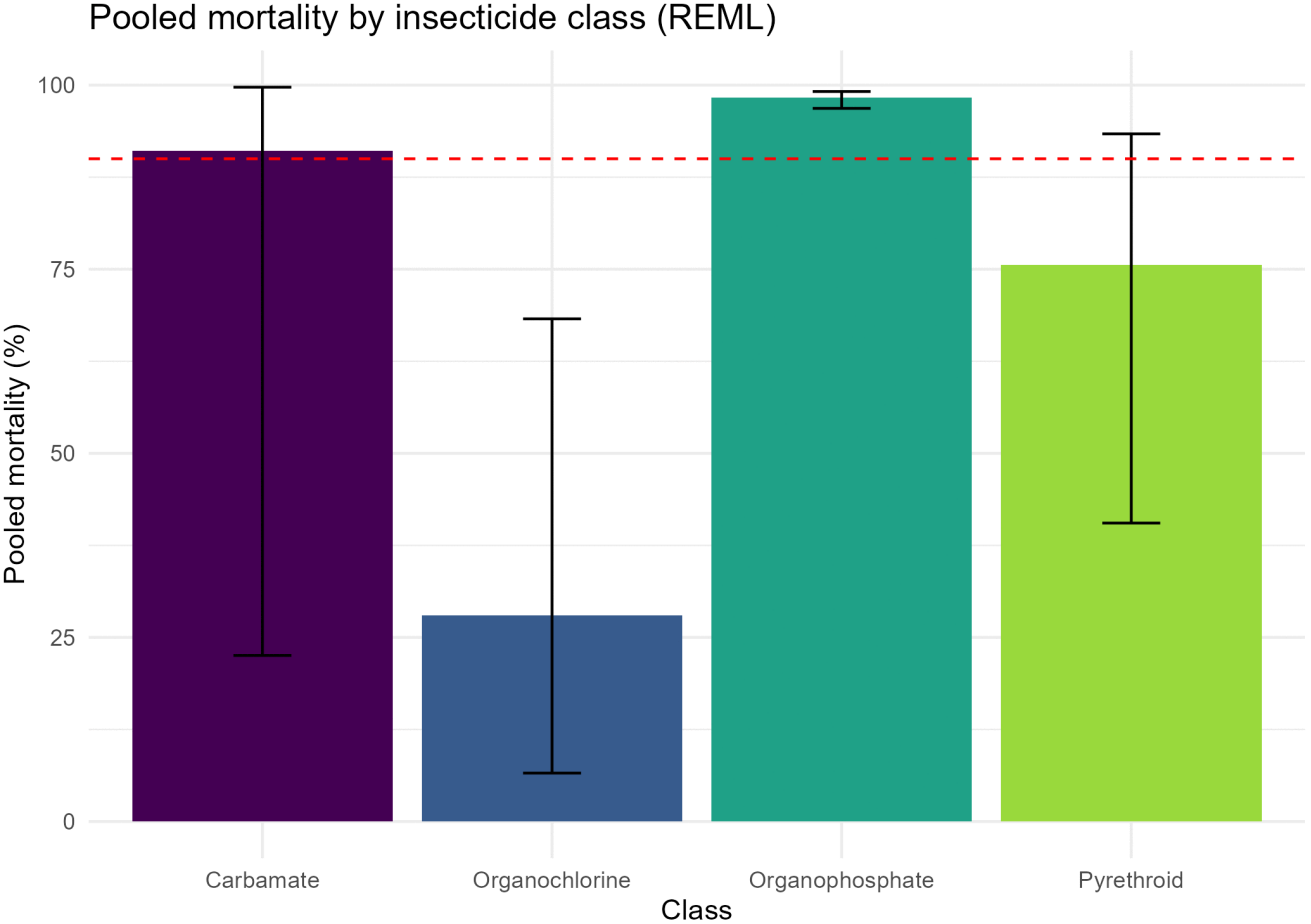
Overall pooled proportional mortality (REML).

Fig 5 presents the forest plot of study-level mortality (%) stratified by insecticide class, with pooled proportional mortality (PPM) estimated using a random-effects model under restricted maximum likelihood (REML) on logit-transformed proportions. The pooled proportional mortality estimates derived from the random‑effects REML model further underscore the heterogeneity of insecticide resistance in Nigerian *Ae. aegypti* populations. Across all insecticide classes, the overall pooled mortality was 79.6% (95% CI: 57.5–91.8), confirming that susceptibility is generally below WHO thresholds and resistance is widespread. Class‑specific estimates revealed striking differences: organophosphates had the highest pooled mortality at 98.3% (95% CI: 96.8–99.1; k = 6; I² = 0%), suggesting overall effectiveness but masking localized resistance, including moderate malathion resistance in Lagos, low‑level pirimiphos‑methyl resistance in Ebonyi and Delta, and established temephos resistance in Kano. Carbamates yielded a pooled mortality of 91.1% (95% CI: 22.6–99.7; k = 4; I² ≈ 96%), reflecting geographic variability, with full susceptibility in Umudike (Abia), borderline resistance in Lagos, and clear resistance in Lokoja (Kogi). Pyrethroids demonstrated a pooled mortality of 75.6% (95% CI: 40.5–93.4; k = 13; I² ≈ 98%), confirming entrenched resistance across multiple states. Organochlorines, represented by DDT, exhibited the lowest pooled mortality at 28.0% (95% CI: 6.6–68.3; k = 7; I² ≈ 98%), consistent with severe resistance nationwide.

These pooled estimates highlight the dominance of pyrethroid and DDT resistance, the variable but concerning emergence of carbamate resistance, and the declining reliability of organophosphates despite their comparatively higher pooled mortality. The high I² values for pyrethroids, carbamates, and DDT reflect substantial between‑study heterogeneity, underscoring the importance of regional surveillance and tailored control strategies. In contrast, the low heterogeneity observed for organophosphates suggests more consistent performance across studies, though temporal data indicate that this effectiveness may not be sustained in the future.

Bar chart showing pooled mortality estimates for *Ae. aegypti* across four insecticide classes: carbamates, organochlorines, organophosphates, and pyrethroids. Bars represent pooled mortality percentages with error bars indicating confidence intervals. The dashed red horizontal line marks the 100% mortality reference threshold. Results highlight comparative efficacy, with carbamates and organophosphates showing the highest pooled mortality, while organochlorines exhibit the lowest.

### 3.9 Leave‑one‑out pooled PPM (%)

**Fig 6:**
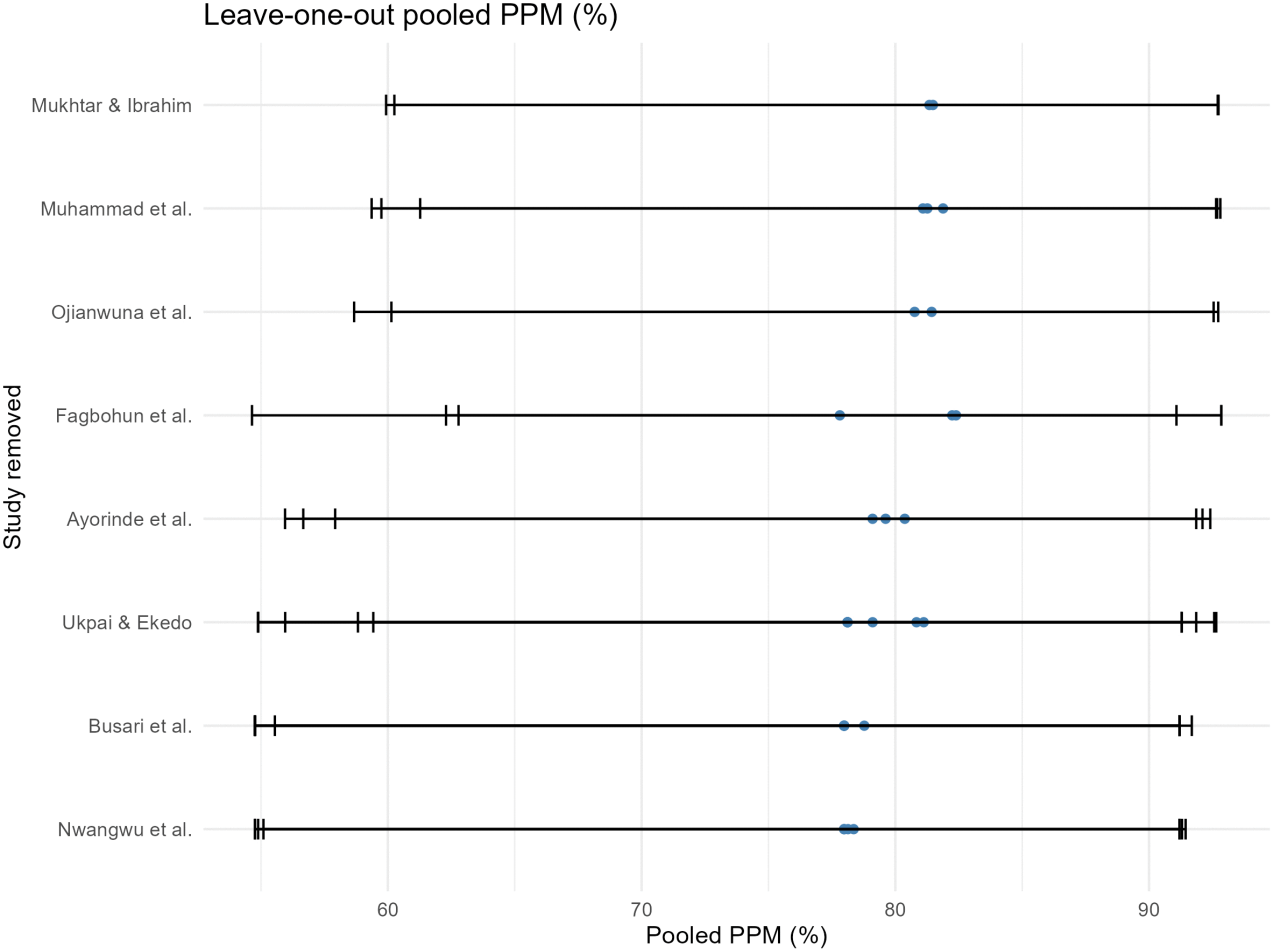
Leave‑one‑out pooled PPM (%).

Fig 6 shows the leave-one-out pooled PPM(%). The robustness of the pooled proportional mortality estimates was evaluated using a leave‑one‑out sensitivity analysis. Sequential exclusion of individual studies produced pooled mortality values ranging from 77.8% to 82.4%, with a median of 79.5%, closely matching the overall pooled estimate of 79.6% (95% CI: 57.5–91.8). The narrow range of recalculated values indicates that the random‑effects REML model was stable and not unduly influenced by any single study. Importantly, omission of high‑impact studies, such as those from Lagos [22], Kano [16], and Delta [25], produced only minor shifts in the pooled estimates, confirming the consistency of the overall resistance pattern.

This stability reinforces the conclusion that pyrethroid and DDT resistance is entrenched nationwide, carbamate susceptibility remains geographically variable, and organophosphates, while currently yielding high pooled mortality, are showing early signs of decline in specific regions. The sensitivity analysis therefore supports the reliability of the pooled estimates while highlighting the importance of continued surveillance to capture emerging resistance trends.

Forest plot showing the effect of sequentially removing individual studies on the pooled percentage mortality (PPM). Each blue dot represents the pooled estimate when the corresponding study is excluded, with horizontal lines indicating the 95% confidence interval. The x‑axis displays pooled PPM (%) and the y‑axis lists the studies removed. This analysis demonstrates the robustness of the overall pooled estimate by assessing the influence of each study on the summary outcome.

### 3.10 Assessment of publication bias and small‑study effects

**Fig 7:**
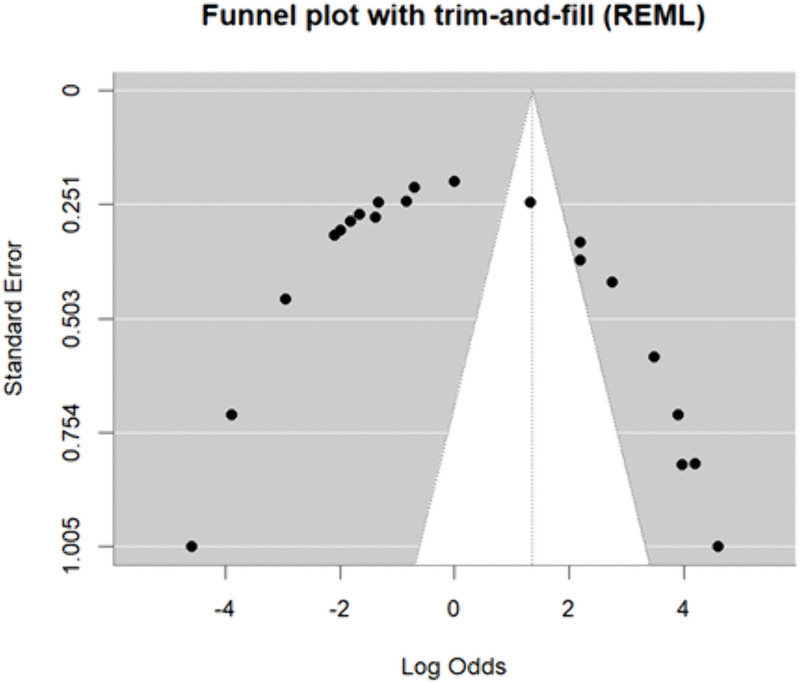
Assessment of Publication Bias and Small‑Study Effects.

Fig 7 illustrates the publication bias and small-study effects. Assessment of publication bias and small‑study effects was conducted using funnel plot visualization and Egger’s regression. The overall pooled proportional mortality was 79.6% (95% CI: 57.5–91.8; k = 30). The funnel plot displayed individual study effects plotted against their standard errors, with the dashed vertical line representing the pooled logit estimate from the REML model. Visual inspection revealed asymmetry, which was confirmed statistically by Egger’s regression (z = 3.68, p = 0.0002), indicating potential small‑study effects.

However, application of the Trim‑and‑Fill procedure did not impute any missing studies, and the pooled estimate remained unchanged, suggesting that the observed asymmetry was not driven by missing data. Instead, the asymmetry likely reflects heterogeneity related to study size, geographic coverage, or methodological differences. For example, smaller studies conducted in single states such as Lagos or Delta often reported extreme resistance values (mortality <30% for DDT, < 90% for pyrethroids), whereas larger regional surveys tended to produce more moderate estimates.

Taken together, these findings suggest that while small‑study effects are present, they do not materially alter the overall pooled estimates. The consistency of the pooled mortality values across sensitivity analyses reinforces the robustness of the conclusion: pyrethroid and DDT resistance is entrenched nationwide, carbamate susceptibility is geographically variable, and organophosphate effectiveness, though currently high, is beginning to decline in certain regions.

Funnel plot of study precision (standard error) against effect size (log odds) used to evaluate potential publication bias in the meta‑analysis. Black dots represent individual study estimates. The central triangular region indicates the expected distribution under symmetry, while the trim‑and‑fill method (REML) adjusts for potentially missing studies to restore balance. The plot provides a visual assessment of small‑study effects and the robustness of the pooled estimates.

### 3.11 Random effects study intercepts (Caterpillar plot)

**Fig 8:**
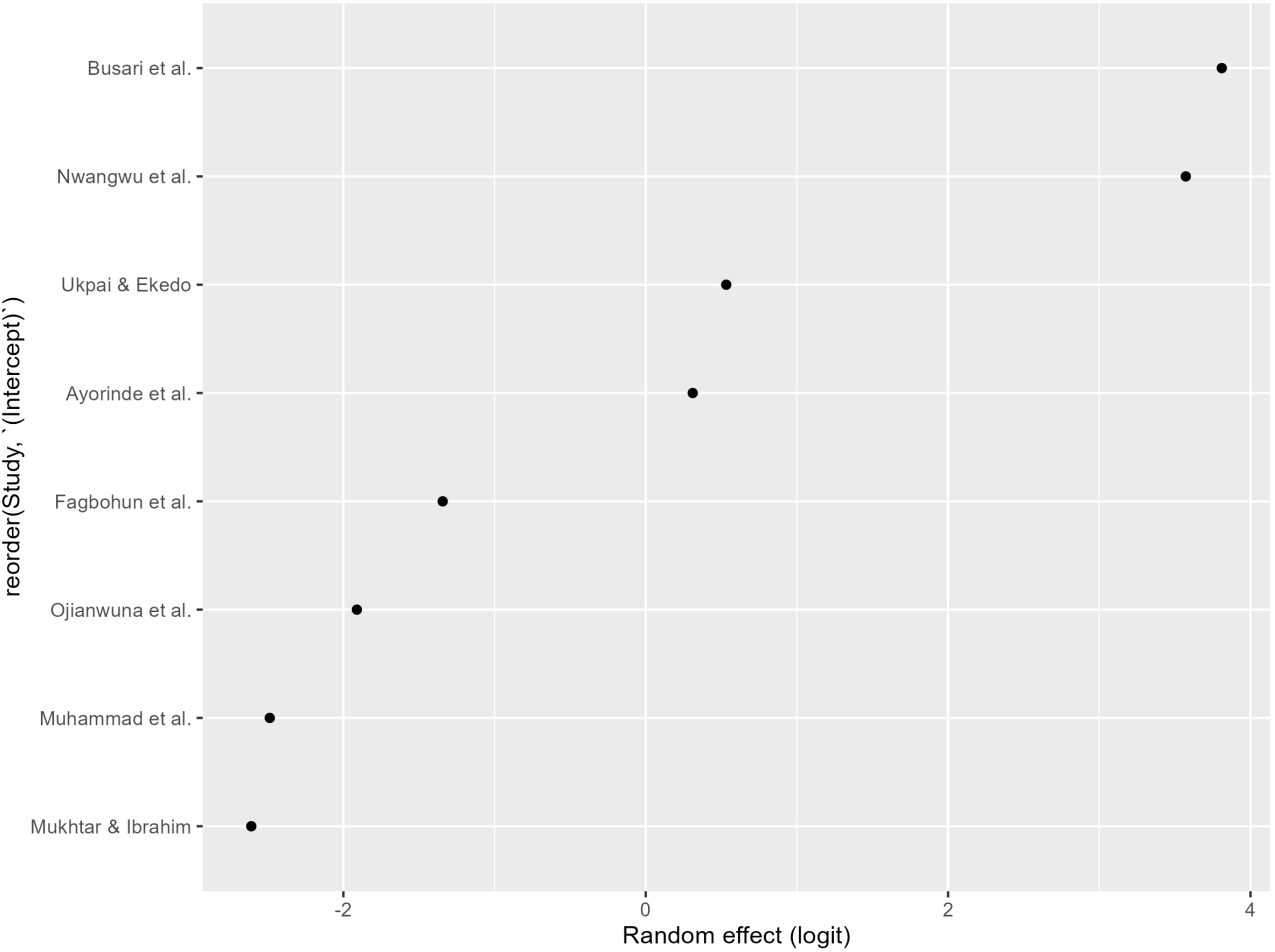
Random Effects Study Intercepts (Caterpillar Plot).

Fig 8 shows the Random Effects Study Intercepts. The caterpillar plot of study‑level random intercepts provided further insight into the heterogeneity underlying insecticide mortality estimates. Each study’s random effect, expressed as a best linear unbiased prediction (BLUP) on the logit scale, demonstrated wide dispersion, with approximate 95% confidence intervals spanning a broad range of values. The mixed‑effects GLMM estimated a between‑study variance (τ²) of 21.49, corresponding to an I² of approximately 99.3%, confirming substantial heterogeneity across the included studies.

The pooled proportional mortality estimated by the GLMM was 94.2% (95% CI: 71.9–99.0), slightly higher than the REML estimate of 79.6%, reflecting the influence of model choice and weighting. The pronounced variability in study intercepts highlights the uneven distribution of resistance across Nigeria: extreme low mortality values for DDT and pyrethroids in Lagos, Delta, and Kano contrasted with high mortality values for carbamates and organophosphates in Abia and Osun. This dispersion underscores the importance of interpreting pooled estimates alongside regional data, as national averages may obscure localized resistance hotspots.

Overall, the caterpillar plot confirms that while pooled models provide useful summary estimates, the underlying heterogeneity is biologically meaningful. Resistance patterns vary sharply by insecticide class and geographic region, reinforcing the need for spatially targeted surveillance and adaptive vector control strategies.

Scatter plot showing study‑specific random effect estimates (logit scale) from the meta‑analysis. Each dot represents the random effect for an individual study. The x‑axis displays the random effect values, while the y‑axis lists the studies. This visualization highlights variability in study‑level contributions to the overall model and helps identify potential outliers or influential studies.

## 4 Discussion

### 4.1 Overall pattern of insecticide resistance

This meta‑analysis confirms a pervasive pattern of insecticide resistance in *Ae. aegypti* populations across Nigeria. The most consistent and concerning findings were the high levels of resistance to pyrethroids and organochlorines. Pooled mortality estimates for pyrethroids (75.6%) and DDT (28.0%) were well below the WHO susceptibility threshold of 90%, corroborating widespread resistance across Lagos, Kano, Delta, and Kogi. The reduced pyrethroid mortality aligns with the high frequency of F1534C kdr mutations detected in Lagos and Kano, while the extremely low DDT mortality reflects both target-site insensitivity and elevated glutathione S‑transferase activity. These results mirror global evidence from Africa, Asia, and Latin America, where pyrethroid resistance has become entrenched due to combined public health and agricultural pesticide pressures. The Nigerian data therefore reinforce the global trend of declining pyrethroid efficacy and confirm that DDT is no longer viable for vector control. Geographic heterogeneity in carbamate mortality (91.1%) corresponds to variable susceptibility observed in Abia (susceptible), Lagos (borderline), and Kogi (resistant). In contrast, the high pooled organophosphate mortality (98.3%) suggests overall effectiveness, but masks emerging resistance to malathion, pirimiphos‑methyl, and temephos in specific regions.

### 4.2 Mechanistic basis of resistance

The mechanistic basis of resistance is strongly linked to both target-site insensitivity and metabolic detoxification. Knockdown resistance (kdr) mutations, particularly F1534C (frequent) and S989P (rare), were consistently detected, with their co‑occurrence documented for the first time in Africa. These mutations impair insecticide binding to the voltage‑gated sodium channel, conferring resistance to both pyrethroids and DDT. In parallel, metabolic resistance mediated by elevated cytochrome P450 monooxygenases and glutathione S‑transferases was confirmed through synergist assays, which partially restored susceptibility when Piperonyl Butoxide (PBO) was applied. The convergence of molecular and metabolic mechanisms underscores the robustness of resistance in Nigerian *Ae. aegypti* populations and explains the multi-insecticide resistance (MIR) profile observed.

### 4.3 Heterogeneity across insecticide classes

In contrast to pyrethroids and DDT, susceptibility to carbamates and organophosphates varied geographically. Carbamates showed full susceptibility in Umudike (Abia), borderline resistance in Lagos, and clear resistance in Lokoja (Kogi), yielding a pooled mortality of 91.1% but with high heterogeneity (I² ≈ 96%). Organophosphates had the highest pooled mortality (98.3%) and low heterogeneity (I² = 0%), suggesting overall effectiveness. However, regional data revealed emerging resistance: moderate malathion resistance in Lagos, low‑level pirimiphos‑methyl resistance in Ebonyi and Delta, and established temephos resistance in Kano, where mortality dropped below 50% by 2020. These findings highlight that while organophosphates currently outperform other classes, their reliability is eroding, and resistance is spreading across regions.

### 4.4 Operational and policy implications

The dominance of multi-insecticide resistance in Nigerian *Ae. aegypti* presents a substantial challenge to vector control programs. Pyrethroids remain the cornerstone of public health interventions, yet their declining efficacy threatens the sustainability of these strategies. The limited effectiveness of carbamates and the emerging resistance to organophosphates further restrict rotation options, a standard resistance management practice. To sustain vector control efficacy, Nigeria must integrate non‑chemical approaches such as Wolbachia‑based strategies, biological control, and environmental management, alongside improved resistance monitoring. Genomic surveillance of kdr mutations and metabolic markers will be critical to anticipate resistance spread and guide adaptive control strategies.

### 4.5 Limitations and future directions

This review was constrained by the relatively small number of eligible studies and the concentration of research in southern Nigeria, particularly Lagos. These gaps reflect the uneven distribution of entomological surveillance capacity across the country rather than weaknesses in the review process itself. The wide confidence intervals observed in pooled estimates highlight substantial heterogeneity and limited precision, especially for temporal predictions, which is an inherent challenge when synthesizing data from diverse study designs and geographic contexts. Importantly, these limitations underscore the urgent need for expanded surveillance in underrepresented northern regions, the adoption of standardized WHO bioassay protocols to ensure comparability across sites, and the integration of genomic and transcriptomic approaches to better characterize resistance mechanisms. Longitudinal monitoring will be essential to track resistance trends over time and to inform adaptive, evidence‑based vector control strategies. By identifying these gaps, the present review provides a roadmap for strengthening future research and surveillance efforts in Nigeria.

The limitations observed in Nigeria mirror challenges faced across many low‑ and middle‑income countries, where surveillance is uneven, resources are concentrated in urban centers, and reliance on WHO bioassays remains the norm. By situating Nigeria’s resistance profile within this broader global context, the findings reinforce the urgent need for coordinated international efforts to standardize monitoring, expand genomic surveillance, and develop adaptive vector control strategies that can respond to rapidly evolving resistance patterns. Thus, while constrained by available data, this review contributes both nationally and globally to the evidence base guiding sustainable arbovirus vector control.

### 4.6 Conclusion

To the best of our knowledge, this systematic review and meta‑analysis provide the first comprehensive synthesis of insecticide resistance in *Ae aegypti* populations across Nigeria. The findings confirm entrenched resistance to pyrethroids and DDT, variable susceptibility to carbamates, and emerging resistance to organophosphates. These patterns are driven by both target-site mutations, such as F1534C, and metabolic detoxification mechanisms, including cytochrome P450 monooxygenases and glutathione S‑transferases.

Despite the limited number of studies and uneven geographic coverage, the pooled estimates highlight a consistent national trend: susceptibility is generally below WHO thresholds, and resistance is widespread. This evidence underscores the urgent need for adaptive vector control strategies that move beyond reliance on pyrethroids and DDT, while strengthening surveillance in underrepresented regions.

Nigeria’s situation reflects broader challenges faced across low‑ and middle‑income countries, where surveillance capacity is uneven and resistance monitoring often depends on WHO bioassays. By situating these findings within the global context, this review contributes actionable evidence for both national policy and international efforts to sustain effective arbovirus vector control. Expanding genomic surveillance, standardizing bioassay protocols, and investing in longitudinal monitoring will be critical to mitigating resistance and safeguarding public health.

## Supporting information

S1 Fig PRISMA_flow_diagram.docx PRISMA 2020 flow diagram summarizing the search and selection process for the systematic review. (DOCX)

S1 Checklist PRISMA_2020_checklist.docx PRISMA 2020 checklist for reporting systematic reviews and meta‑analyses. (DOCX)

S1 File Registered_protocol.pdf Registered protocol for the systematic review and meta‑analysis (https://doi.org/10.17605/OSF.IO/CTUH8). (PDF)

S2 File Eligibility_Criteria.docx Modified PECO framework defining inclusion and exclusion criteria for relevant studies. (DOCX)

S3 File Result_Table_Overview.docx Summary table of included studies with pooled mortality estimates and key notes. (DOCX)

S1 Table Study screening and exclusion log. (XLSX)

S2 Table Study extraction form and extracted variables. (XLSX)

S3 Table Risk of bias assessment for included studies. Adapted Joanna Briggs Institute checklist with item‑level judgments and overall risk of bias for each included study.
(DOCX)
