# Supplementary material for: Systematic review and meta-analysis of insecticide resistance status and mechanisms in the arbovirus vector Aedes aegypti from Nigeria
Source: PLoS Negl Trop Dis. 2026 Jun 15;20(6):e0014421. doi: 10.1371/journal.pntd.0014421 (PMC13278583; doi:10.1371/journal.pntd.0014421)
Supplement: S1 Fig — (DOCX) [file pntd.0014421.s001.docx]

Records identified from

Databases (n =130)

Registers (n = 0)

**Identification of studies via databases and registers (Scopus, Google Scholar, PubMed, Web of Science, AJOL, VectorBase)**

Reports not retrieved

(n = 0)

Reports sought for retrieval

(n =85)

Reports assessed for eligibility

(n = 85)

Reports excluded:

Not aedes n=32

No clear geographic data n= 10

Review articles, Commentaries, Editorials, Case reports n= 17

Insufficient methodological details or missing outcomes n=17

**es only (n = 12)**

• • **No clear geographic data for Nigeria (n = 10)**

• • **Review articles, commentaries, editorials, or case reports (n = 15)**

• • **Insufficient methodological details or missing outcome data (n = 19)**

•

Studies included in review

(n =9)

Reports of included studies

(n =0)

**Included**

Records removed *before screening*:

Duplicate records removed (n =45)

Records marked as ineligible by automation tools (n =0)

Records removed for other reasons (n =0)

Records screened

(n =85)

Records excluded**

(n =76)

**Identification**

**Screening**

*Consider, if feasible to do so, reporting the number of records identified from each database or register searched (rather than the total number across all databases/registers).

**If automation tools were used, indicate how many records were excluded by a human and how many were excluded by automation tools.

Source: Page MJ, et al. BMJ 2021;372:n71. doi: 10.1136/bmj.n71.

This work is licensed under CC BY 4.0. To view a copy of this license, visit <https://creativecommons.org/licenses/by/4.0/>
