# Supplementary material for: Systematic review and meta-analysis of insecticide resistance status and mechanisms in the arbovirus vector Aedes aegypti from Nigeria
Source: PLoS Negl Trop Dis. 2026 Jun 15;20(6):e0014421. doi: 10.1371/journal.pntd.0014421 (PMC13278583; doi:10.1371/journal.pntd.0014421)
Supplement: S1 File — (PDF) [file pntd.0014421.s003.pdf]

Study Information

Hypotheses

Resistance to pyrethroids in Aedes aegypti populations in Nigeria is significantly higher than resistance to carbamates and organophosphates (directional; predicted lower mortality in pyrethroid bioassays). Resistance to organochlorines (DDT) is greater than resistance to all other insecticide classes (directional; predicted lowest mortality in DDT bioassays). The frequency of knockdown resistance (kdr) mutations, including F1534C and S989P, is positively associated with pyrethroid and DDT resistance levels (directional; predicted inverse relationship between mutation frequency and mortality). Elevated metabolic enzyme activity, particularly cytochrome P450 monooxygenases and glutathione S-transferases, is associated with reduced susceptibility to pyrethroids and DDT (directional; predicted lower mortality in populations with high enzyme activity). The effect of insecticide class on mortality rates is moderated by geographic region (directional interaction; predicted higher carbamate and organophosphate resistance in northern regions compared to southern regions). Finally, resistance prevalence is associated with year of study (non-directional; predicted temporal variation in resistance levels).

Design Plan

Study type

Meta-Analysis - A systematic review of published studies.

Blinding

No blinding is involved in this study.

Is there any additional blinding in this study?

No data

Study design

My study is a systematic review and meta-analysis of insecticide resistance in Aedes aegypti mosquitoes in Nigeria. It follows the PRISMA 2020 guidelines and was registered a priori on the Open Science Framework (OSF). The design involves a comprehensive search across multiple databases (PubMed, Scopus, Web of Science, Google Scholar, AJOL, VectorBase), screening of titles and abstracts, and application of strict inclusion and exclusion criteria based on a modified PECO framework (Population, Exposure, Comparator, Outcome). Eligible studies were original research articles reporting bioassay mortality rates, genetic resistance markers (such as kdr mutations), or metabolic enzyme activity in field-collected Aedes aegypti populations from Nigeria. Studies on other mosquito species, laboratory-only populations, or lacking methodological detail were excluded. Data extraction was standardized, capturing geographic location, insecticide class, mortality rates, and resistance mechanisms. Risk of bias was assessed independently by two reviewers using the Joanna Briggs Institute Critical Appraisal Checklist for Prevalence Studies, ensuring methodological rigor. For the meta-analysis, pooled proportional mortality was estimated using random-effects models (REML), with transformations applied to

prevalence data. Heterogeneity was quantified using I^2 and τ^2 statistics, and subgroup analyses explored differences by insecticide class and geographic region. Publication bias was assessed with funnel plots and Egger's regression.

No files selected

Randomization

No data

Sampling Plan

Existing Data

Registration prior to analysis of the data

Explanation of existing data

For this systematic review and meta-analysis, the data to be used are drawn exclusively from previously published studies identified through the literature search. To ensure that I remain unaware of any patterns or summary statistics prior to formal extraction and analysis, access to the data has been limited to the published articles themselves without any pre-compiled datasets or prior aggregated results. Screening and selection were conducted using titles and abstracts only, without examining outcome data until eligibility was confirmed. Data extraction is performed using a standardized form, and only after inclusion decisions have been finalized. No preliminary analyses or summary statistics of the included studies were reviewed in advance, and the extraction process is carried out independently by two reviewers to minimize bias. In this way, I have avoided exposure to any pre-analyzed datasets or informal summaries, ensuring that the statistical patterns and pooled estimates will only emerge during the formal meta-analysis stage.

Data collection procedures

This study does not involve human subjects. Data collection will be based entirely on published literature retrieved through a systematic search of electronic databases including PubMed, Scopus, Web of Science, Google Scholar, African Journals Online (AJOL), and VectorBase. The search strategy will employ combinations of keywords such as Aedes aegypti, Aedes albopictus, insecticide resistance, Nigeria, and related terms, using Boolean operators to maximize coverage. All records will be imported into reference management software, duplicates removed, and titles and abstracts screened for relevance. Full texts of potentially eligible studies will then be reviewed against predefined criteria. The inclusion criteria are original research articles published in English that report insecticide resistance in Aedes aegypti or Aedes albopictus populations from Nigeria. Eligible studies must provide primary data through bioassays (mortality rates), genetic resistance markers (such as kdr mutations), or metabolic enzyme activity assays. Studies that include clear geographic information and adhere to WHO bioassay protocols or equivalent methodologies will be prioritized. Exclusion criteria are studies focusing solely on non-Aedes mosquito species, review articles, commentaries, editorials, case reports, and studies lacking sufficient methodological detail or primary outcome data. Laboratory-only populations without field relevance and studies without quantitative resistance outcomes will also be excluded. The data

gathering process will span the period from January 2000 to December 2025, covering all eligible publications within this timeframe. Data extraction will be performed using a standardized form to capture study characteristics, geographic location, insecticide class tested, mortality rates, and resistance mechanisms. Two reviewers will independently extract data and assess risk of bias using the Joanna Briggs Institute Critical Appraisal Checklist for Prevalence Studies, with disagreements resolved by consensus. This process ensures that only high-quality, relevant data are included in the synthesis and subsequent meta-analysis.

No files selected

Sample size

The unit of analysis is the study-level bioassay arm reporting 24-hour mortality for a specific insecticide class in field-collected Aedes aegypti from Nigeria. The systematic review includes 9 primary studies, which together contribute approximately 30 study arms/effects to the meta-analysis (e.g., organophosphate k = 6, carbamate k = 4, pyrethroid k = 13, organochlorine/DDT k = 7). This yields a clustered, multilevel structure with multiple class-specific effects nested within studies, and subgrouping by geographic region when applicable. Per-unit (arm) sample size requirements follow WHO guidance and the JBI criteria used in this review: at least 100 mosquitoes tested per bioassay/insecticide concentration, with studies required to report the number exposed and mortality outcomes. Where studies present multiple concentrations or sites, each eligible arm is treated as a separate unit with its reported N and mortality, and pooled using random-effects models.

Sample size rationale

No data

Stopping rule

No data

Variables

Manipulated variables

The primary variable is insecticide class, with four levels corresponding to the major public health insecticide groups: pyrethroids (e.g., permethrin, deltamethrin), organochlorines (DDT), carbamates (e.g., bendiocarb), and organophosphates (e.g., malathion, primiphos-methyl). The outcome variable is mortality rate (%) at 24 hours post-exposure, which serves as the measure of susceptibility or resistance. Secondary variables include mechanism of resistance, with two categories: target-site insensitivity (presence of kdr mutations such as F1534C, S989P, V1016I/L) and metabolic resistance (elevated activity of detoxifying enzymes such as cytochrome P450 monooxygenases and glutathione S-transferases). Another variable is geographic region, categorized by Nigerian geopolitical zones (North-West, North-Central, South-West, South-East, South-South). Year of study is also included as a continuous variable to assess temporal trends. Thus, while no variables are manipulated experimentally, the meta-analysis evaluates differences

across insecticide classes, mechanisms, regions, and time, treating these as natural grouping or moderator variables within the pooled analysis.

No files selected

Measured variables

In this systematic review and meta-analysis, the primary outcome variable is the 24-hour post-exposure mortality rate (%) of Aedes aegypti following insecticide bioassays. This measure is used to classify populations as susceptible or resistant according to WHO thresholds. The key predictor variable is insecticide class, with four defined levels: pyrethroids (permethrin, deltamethrin), organochlorines (DDT), carbamates (bendiocarb), and organophosphates (malathion, primiphos-methyl). Additional measured covariates include: Resistance mechanisms: presence and frequency of knockdown resistance (kdr) mutations (F1534C, S989P, V1016I/L) and elevated metabolic enzyme activity (cytochrome P450 monooxygenases, glutathione S-transferases, esterases). Geographic region: Nigerian geopolitical zones (North-West, North-Central, South-West, South-East, South-South). Year of study: publication year of each included study, treated as a continuous variable to assess temporal trends. Sample size: number of mosquitoes tested per bioassay arm, used to weight effect sizes in the meta-analysis.

No files selected

Indices

No data

No files selected

Analysis Plan

Statistical models

Statistical models Model overview Primary effect size: 24-hour mortality proportion per bioassay arm, transformed to logit scale with continuity correction. y_i = logit (x_i + 0.5 n_i + 1) , v_i = 1 (x_i + 0.5) + 1 ($n_i - x_i$ + 0.5) Estimator: Random-effects meta-analysis with REML, inverse-variance weighting. Clustering: Random intercept for study to account for multiple arms per study. Software: R (metafor). Core functions: rma.uni for class-level models; rma.mv for clustered/multilevel; robust variance optional via clubSandwich for sensitivity. Hypothesis-specific models H1: Pyrethroid vs carbamate and organophosphate Model: Mixed-effects meta-regression with insecticide class as a categorical predictor; random intercept for study. $y_{ij} = \beta_0 + \beta_1$ Class Pyrethroid + β_2 Class Carbamate + β_3 Class OrgPhos + $u_j + \epsilon_{ij}$ Tests: Planned contrasts (Wald tests) of pyrethroid vs carbamate and pyrethroid vs organophosphate on the logit scale; back-transform to percent mortality. Follow-ups: Pairwise contrasts among all classes with Holm adjustment; report τ^2, I^2, Q. H2: DDT vs other classes Model: As above, with contrast DDT vs pooled non-DDT classes. H_0 : β DDT = β Pyrethroid + β Carbamate + β OrgPhos 3 Tests: Single planned contrast DDT vs non-DDT; secondary pairwise DDT vs each class. H3: kdr mutation frequency association (pyrethroid, DDT) Model: Meta-regression with continuous predictors for mutation frequency (%), stratified by class or with interaction. $y_{ij} = \beta_0 + \beta_1$ Freq

$_{1534C} + \beta_2 \text{Freq}_{989P} + \beta_3 \text{Class} + \beta_4 (\text{Freq} \times \text{Class}) + u_j + \epsilon_{ij}$ Tests: Wald tests for slopes (β1, β2) within pyrethroid and DDT; interaction terms (β4) to confirm class-specific associations. Sensitivity: Standardize frequencies; check collinearity; fit separate models per mutation if needed. H4: Metabolic enzyme activity association Model: Meta-regression with enzyme activity as continuous (fold-change vs susceptible) or binary (elevated vs baseline); include class and interaction. $y_{ij} = \beta_0 + \beta_1 \text{P450 activity} + \beta_2 \text{GST activity} + \beta_3 \text{Class} + \beta_4 (\text{Enzyme} \times \text{Class}) + u_j + \epsilon_{ij}$ Tests: Wald tests for β1, β2; interactions to assess class-specific effects. Alternative: Use PBO effect size (mortality with PBO minus without) as predictor in sensitivity analysis. H5: Geographic moderation (region × class) Model: Meta-regression with region (North vs South or geopolitical zones) and class, plus interaction. $y_{ij} = \beta_0 + \beta_1 \text{Region} + \beta_2 \text{Class} + \beta_3 (\text{Region} \times \text{Class}) + u_j + \epsilon_{ij}$ Tests: Simple effects of region within each class; contrasts North vs South for carbamate and organophosphate; report interaction β3. H6: Temporal trend (year and class × year) Model: Meta-regression with year (centered) and class, plus interaction for class-specific slopes. $y_{ij} = \beta_0 + \beta_1 \text{Year} + \beta_2 \text{Class} + \beta_3 (\text{Year} \times \text{Class}) + u_j + \epsilon_{ij}$ Tests: Wald tests for β1 (overall trend) and β3 (class-specific trends); report predicted 2025 mortality per class with back-transformation and 95% CIs. Subgroup analyses, contrasts, and follow-ups Subgroups: By insecticide class; by region; by assay adherence to WHO DD (yes/no). Contrasts: Pre-specified pairwise contrasts among classes; region contrasts within class; mutation/enzyme slope comparisons across classes. Multiple testing: Holm or Benjamini–Hochberg adjustments for families of contrasts. Influence/sensitivity: Leave-one-out; dfbetas; re-fit with different transformations (double-arcsine) for robustness; robust variance estimation for clustering. Publication bias and small-study effects Funnel plot: On logit effects vs SE; Egger's regression (intercept test). Trim-and-fill: Applied if asymmetry detected; compare pooled estimates pre/post. Replicable specification (metafor examples) Random-effects by class: rma.mv(yi, vi, mods = ~ Class, random = ~ 1 | StudyID, method = "REML") Interaction model (region × class): rma.mv(yi, vi, mods = ~ Class*Region, random = ~ 1 | StudyID, method = "REML") Temporal trend: rma.mv(yi, vi, mods = ~ Class*YearCentered, random = ~ 1 | StudyID, method = "REML") Mechanism models: rma.mv(yi, vi, mods = ~ ClassFreq1534C + ClassFreq989P) and rma.mv(yi, vi, mods = ~ ClassP450 + ClassGST) Contrasts: Use linearHypothesis or custom contrast matrices; back-transform predictions with plogis for percent mortality.

No files selected

Transformations

No data

Inference criteria

No data

Data exclusion

No data

Missing data

No data

Exploratory analysis

No data

Other

Other

No data
