# Supplementary material for: Systematic review and meta-analysis of insecticide resistance status and mechanisms in the arbovirus vector Aedes aegypti from Nigeria
Source: PLoS Negl Trop Dis. 2026 Jun 15;20(6):e0014421. doi: 10.1371/journal.pntd.0014421 (PMC13278583; doi:10.1371/journal.pntd.0014421)
Supplement: S2 File — (DOCX) [file pntd.0014421.s004.docx]

**Eligibility Criteria**

We utilized a modified PECO (Population, Exposure, Comparator, Outcome) framework to define the inclusion and exclusion criteria for relevant studies.

| Component | Criteria |
| --- | --- |
| Population (P) | Studies involving field-collected populations of Aedes aegypti from Nigeria only. |
| Exposure (E) | Bioassays conducted using WHO discriminating doses (DD) of common public health insecticides, including Pyrethroids (e.g., Permethrin, Deltamethrin), Organochlorines (DDT), Organophosphates (e.g., Malathion, Pirimiphos-methyl), and Carbamates (e.g., Bendiocarb). |
| Comparator (C) | Studies using a susceptible reference strain or a negative control group (e.g., filter paper impregnated with oil only). |
| Outcome (O) | Studies reporting quantitative data on: Insecticide susceptibility status (24-hour post-exposure mortality rates) or Resistance mechanisms (*kdr* mutation frequencies or biochemical enzyme activity levels—Cytochrome P450 monooxygenases, Glutathione S-transferases, and non-specific esterases). |
| Study Design/Time | Original research articles, grey literature, and dissertations published from January 2000 to December 2025. |
