# Supplementary material for: Systematic review and meta-analysis of insecticide resistance status and mechanisms in the arbovirus vector Aedes aegypti from Nigeria
Source: PLoS Negl Trop Dis. 2026 Jun 15;20(6):e0014421. doi: 10.1371/journal.pntd.0014421 (PMC13278583; doi:10.1371/journal.pntd.0014421)
Supplement: S3 File — (DOCX) [file pntd.0014421.s005.docx]

**Result Table Overview**

| \| **Publication Year** \| **Authors** \| **Location (State)** \| **Geo‑Political Zone** \| **Target Species** \| **Insecticide Classes Evaluated** \| **Key Study Notes** \| **Class‑Level Pooled Mortality (%)** \| **95% CI** \|  \| **I² (%)** \| \| --- \| --- \| --- \| --- \| --- \| --- \| --- \| --- \| --- \| --- \| --- \| \| **2015** \| Ayorinde et al. \| Lagos \| South‑West \| *Ae. aegypti* \| Organochlorines, Pyrethroids \| Severe DDT & pyrethroid resistance \| DDT: 28.0 \| 6.6–68.3 \|  \| ≈98 \| \| **2018** \| Mkpola & Mathias \| Umudike (Abia) \| South‑East \| *Ae. aegypti* \| Organochlorines, Pyrethroids, Carbamates, Organophosphates \| Susceptible to carbamates/organophosphates; resistant to DDT \| Carbamates: 91.1 \| 22.6–99.7 \|  \| ≈96 \| \| **2020** \| Fagbohun, Idowu, Olakiigbe et al. \| Lagos \| South‑West \| *Ae. aegypti* \| Organochlorines, Pyrethroids, Carbamates \| Variable carbamate susceptibility; strong pyrethroid/DDT resistance \| Pyrethroids: 75.6 \| 40.5–93.4 \|  \| ≈98 \| \| **2021** \| Fagbohun et al. \| Lagos \| South‑West \| *Ae. aegypti* \| Pyrethroids (kdr mutations) \| High F1534C frequency; rare S989P; co‑occurrence reported \| Pyrethroids: 75.6 \| 40.5–93.4 \|  \| ≈98 \| \| **2022** \| Mukhtar & Ibrahim \| Kano \| North‑West \| *Ae. aegypti* \| Organochlorines, Pyrethroids, Carbamates, Organophosphates \| Severe pyrethroid/DDT resistance; temephos resistance established \| Organophosphates: 98.3 \| 96.8–99.1 \|  \| 0 \| \| **2024** \| Ojianwuna et al. \| Delta \| South‑South \| *Ae. aegypti* \| Organochlorines, Pyrethroids \| Resistance to lambda‑cyhalothrin; PBO restored susceptibility \| Pyrethroids: 75.6 \| 40.5–93.4 \|  \| ≈98 \| \| **2024** \| Sani Sade Muhammad et al. \| Lokoja (Kogi) \| North‑Central \| *Ae. aegypti* \| Organochlorines, Pyrethroids, Carbamates \| Resistant to carbamates, pyrethroids, and DDT \| Carbamates: 91.1 \| 22.6–99.7 \|  \| ≈96 \| \| **2025** \| Busari et al. \| Osun \| South‑West \| *Ae. aegypti* \| Organochlorines, Pyrethroids \| Fully susceptible to all tested insecticides \| All classes: ~100 \| – \|  \| – \| \| **2025** \| Nwangwu et al. \| Southern Nigeria (Regional) \| South‑South / South‑East / South‑West \| *Ae. aegypti* & spp. \| Organochlorines, Pyrethroids, Carbamates, Organophosphates \| Regional survey; widespread pyrethroid/DDT resistance; variable carbamate/organophosphate susceptibility \| Mixed \| – \|  \| – \| |
| --- | --- | --- | --- | --- | --- | --- | --- | --- | --- | --- | --- | --- | --- | --- | --- | --- | --- | --- | --- | --- | --- | --- | --- | --- | --- | --- | --- | --- | --- | --- | --- | --- | --- | --- | --- | --- | --- | --- | --- | --- | --- | --- | --- | --- | --- | --- | --- | --- | --- | --- | --- | --- | --- | --- | --- | --- | --- | --- | --- | --- | --- | --- | --- | --- | --- | --- | --- | --- | --- | --- | --- | --- | --- | --- | --- | --- | --- | --- | --- | --- | --- | --- | --- | --- | --- | --- | --- | --- | --- | --- | --- | --- | --- | --- | --- | --- | --- | --- | --- | --- | --- | --- | --- | --- | --- | --- | --- | --- | --- | --- |

**S4 Data. Extracted study-level mortality dataset used for meta-analysis of insecticide resistance in Nigerian *Aedes aegypti* populations.**
