# Supplementary material for: Systematic review and meta-analysis of insecticide resistance status and mechanisms in the arbovirus vector Aedes aegypti from Nigeria
Source: PLoS Negl Trop Dis. 2026 Jun 15;20(6):e0014421. doi: 10.1371/journal.pntd.0014421 (PMC13278583; doi:10.1371/journal.pntd.0014421)
Supplement: S3 Table — Adapted Joanna Briggs Institute checklist with item‑level judgments and overall risk of bias for each included study. (DOCX) [file pntd.0014421.s008.docx]

**S4 TABLE RISK OF BIAS ASSESSMENT**

| **S/N** | **Study ID** | **Sample frame appropriate?** | **Sampling method appropriate?** | **Sample size adequate?** | **Species described in detail?** | **Data collected directly?** | **WHO methodology adhered?** | **Analysis appropriate?** | **Confounders addressed?** | **Prevalence + CI reported?** | **Overall Risk of Bias** |
| --- | --- | --- | --- | --- | --- | --- | --- | --- | --- | --- | --- |
| 1 | Muhammad et al., 2024 (Lokoja, Kogi State) | Yes | Yes | Yes | Yes – morphological only | Yes | Yes | Yes | Yes | No | **Moderate** |
| 2 | Fagbohun et al., 2020 (Lagos State, metabolic resistance) | Yes – 4 LGAs sampled | Yes – larvae collected from peri-domestic habitats, reared to adults | Yes – ≥100 per insecticide per site | Yes –morphologically | Yes – bioassays + biochemical assays performed directly | Yes – WHO tube bioassays, discriminating doses | Yes – WHO thresholds, probit regression, CI reported | Yes – age, feeding, insectary conditions controlled | Yes – mortality % and CI reported | **Low** |
| 3 | Nwangwu et al., 2025 (Ebonyi State, Gigabyte) | Yes | Yes | Yes | Yes – morph + molecular (Ae. luteocephalus) | Yes | Yes | Yes | Yes | Yes | **Low** |
| 4 | Busari et al., 2025 (Osun State, Tropical Medicine and Health) | Yes | Yes | Yes | Yes – morphological only | Yes | Yes | Yes | Yes | Yes | **Low** |
| 5 | Mukhtar & Ibrahim, 2022 (Northern Nigeria, Insects) | Yes | Yes | Yes | Yes – morph + molecular (kdr genotyping) | Yes | Yes | Yes | Yes | Yes | **Low** |
| 6 | Ukpai & Ekedo, 2018 (Umudike, Abia State) | Yes | Yes | Yes | Yes – morphological only | Yes | Yes | Yes | No | No | **Moderate** |
| 7 | Ayorinde et al., 2015 (Farm vs. Nonfarm sites, Lagos State) | Yes | Yes | Yes | Yes – morphological only | Yes | Yes | Yes | Yes | Yes | **Low** |
| 8 | Ojianwuna et al., 2024 (Delta State, PLOS ONE) | Yes | Yes | Yes | Yes – morphological only | Yes | Yes | Yes | Yes | Yes | **Low** |
| 9 | Fagbohun et al., 2021 (Ikorodu, Lagos — kdr mutations) | Yes | Yes | Yes | Yes – morph + molecular (F1534C, S989P, V1016G) | Yes | Yes | Yes | Yes | Yes | **Low** |

**RISK OF BIAS CHECKLIST ASSESSMENT**

**Muhammad et al., 2024 – Risk of Bias Assessment**

1. **Sample frame appropriate?** ✔ Yes — mosquitoes collected from Adankolo Campus, Federal University Lokoja, a relevant endemic site in Kogi State.
2. **Sampling method appropriate?** ✔ Yes — larvae collected from abandoned tyres and reared to adults under insectary conditions. Standard WHO approach.
3. **Sample size adequate?** ✔ Yes — 4 replicates × 25 mosquitoes per insecticide = 100 per test, meeting WHO minimum.
4. **Species described in detail?** ✔ Yes — *Aedes aegypti* identified morphologically using Rueda (2004) keys. No molecular confirmation, but clear morphological description.
5. **Data collected directly?** ✔ Yes — WHO bioassays and larval tests performed directly on collected mosquitoes.
6. **WHO methodology adhered?** ✔ Yes — WHO tube bioassays with discriminating doses (DDT 4%, bendiocarb 0.1%, alpha‑cyhalothrin). Mortality thresholds interpreted per WHO 2016.
7. **Analysis appropriate?** ✔ Yes — mortality rates reported, probit analysis used for LC values, ANOVA applied. WHO thresholds correctly applied.
8. **Confounding factors addressed?** ✔ Yes — mosquito age (2–4 days old), insectary conditions (27–30 °C, 75–80% RH), feeding controlled.
9. **Prevalence measure + CI reported?** ✖ No — mortality percentages reported, but confidence intervals not provided for bioassay outcomes (though probit LC values were given).

**Overall Risk of Bias: Moderate**

- Strengths: WHO-standard bioassays, adequate sample size, controlled conditions, statistical analysis.
- Weaknesses: No CI reporting for mortality outcomes, species identification morphological only.

**Nwangwu et al., 2025 – Risk of Bias Assessment**

1. **Sample frame appropriate?** ✔ Yes — mosquitoes collected from three rural communities in Izzi LGA, Ebonyi State (Oyege, Ndiezoke, Offerekpe, Inyimagu). Relevant and representative rural sites.
2. **Sampling method appropriate?** ✔ Yes — larvae and pupae collected from natural (plant axils) and artificial containers (household water storage, discarded containers), plus ovitraps. Systematic and diverse sampling.
3. **Sample size adequate?** ✔ Yes — WHO tube and CDC bottle bioassays used 4 replicates × 25 females per insecticide = 100 per test, meeting WHO minimum.
4. **Species described in detail?** ✔ Yes — morphological identification for all species (*Ae. aegypti*, *Ae. albopictus*, *Ae. luteocephalus*, *Ae. simpsoni complex*). Molecular confirmation performed for *Ae. luteocephalus* (ITS1 sequencing).
5. **Data collected directly?** ✔ Yes — bioassays and biochemical assays performed directly on collected mosquitoes.
6. **WHO methodology adhered?** ✔ Yes — WHO tube tests (pyrethroids, pirimiphos-methyl, DDT) and CDC bottle bioassays (clothianidin, chlorfenapyr) followed standard protocols.
7. **Analysis appropriate?** ✔ Yes — mortality thresholds interpreted using WHO criteria (≥98% susceptible, 90–97% possible resistance, <90% resistant). Statistical tests (t-tests, ANOVA) applied, with 95% CI reported.
8. **Confounding factors addressed?** ✔ Yes — mosquito age (3–5 days old), insectary conditions (27 °C ± 2, RH 80% ± 10, 12:12 light-dark cycle) controlled and reported.
9. **Prevalence measure + CI reported?** ✔ Yes — mortality percentages reported, confidence intervals provided for enzyme assays and bioassay outcomes.

**Overall Risk of Bias: Low**

- Strengths: Multi-site rural sampling, adequate sample size, WHO-standard bioassays, CI reporting, molecular confirmation for one species, biochemical assays included.
- Weaknesses: None significant; comprehensive methods and reporting.

**Busari et al., 2025 – Risk of Bias Assessment**

1. **Sample frame appropriate?** ✔ Yes — larval surveys across rural Ejigbo LGA, Osun State, with mapping of breeding habitats. Relevant and representative.
2. **Sampling method appropriate?** ✔ Yes — systematic larval sampling from multiple habitat types (gutters, pools, containers, tires, drains). Standard WHO approach.
3. **Sample size adequate?** ✔ Yes — 3500 larvae collected, 600 adults reared, 100 mosquitoes per insecticide per bioassay (WHO minimum met).
4. **Species described in detail?** ✔ Yes — *Aedes aegypti* and *Ae. albopictus* identified morphologically using standard taxonomic keys. No molecular confirmation, but clear morphological description.
5. **Data collected directly?** ✔ Yes — WHO tube bioassays performed directly on reared adults.
6. **WHO methodology adhered?** ✔ Yes — WHO tube bioassays with discriminating doses (permethrin 0.75%, deltamethrin 0.05%, alpha‑cypermethrin 0.05%, pirimiphos‑methyl 0.25%). Mortality thresholds interpreted per WHO 2022 manual.
7. **Analysis appropriate?** ✔ Yes — mortality percentages calculated, chi‑square tests applied, 95% confidence intervals reported.
8. **Confounding factors addressed?** ✔ Yes — mosquito age (2–3 days old), insectary conditions controlled, feeding standardized.
9. **Prevalence measure + CI reported?** ✔ Yes — mortality percentages with 95% CI reported (Table 2).

**Overall Risk of Bias: Low**

- Strengths: Large sample size, systematic sampling, WHO-standard bioassays, CI reporting, controlled conditions.
- Weaknesses: Species identification morphological only, but acceptable given context.

**Mukhtar & Ibrahim, 2022 – Risk of Bias Assessment**

1. **Sample frame appropriate?** ✔ Yes — mosquitoes collected from multiple sites (BUK/Kano and Pantami, Gombe), representing northern Nigeria.
2. **Sampling method appropriate?** ✔ Yes — larvae and pupae collected systematically from breeding containers, tyres, stagnant water, reared under insectary conditions.
3. **Sample size adequate?** ✔ Yes — large numbers tested: 540 larvae per year for larval bioassays, 2700 adults for WHO tube bioassays. Meets and exceeds WHO minimum.
4. **Species described in detail?** ✔ Yes — *Aedes aegypti* identified morphologically with pictorial keys; molecular genotyping performed for kdr mutations (F1534C, V1016G).
5. **Data collected directly?** ✔ Yes — bioassays, synergist assays, cone tests, and molecular genotyping performed directly on collected mosquitoes.
6. **WHO methodology adhered?** ✔ Yes — WHO tube bioassays and larval bioassays followed standard discriminating concentrations; cone bioassays with PermaNet®3.0 also used.
7. **Analysis appropriate?** ✔ Yes — probit analysis for LC50/LC90, chi‑square tests, odds ratios for genotype–phenotype associations, confidence intervals reported.
8. **Confounding factors addressed?** ✔ Yes — mosquito age (3–5 days old), insectary conditions (27 °C ± 2, RH 75% ± 10, 12:12 light–dark cycle) controlled and reported.
9. **Prevalence measure + CI reported?** ✔ Yes — mortality percentages, LC values with 95% CI, resistance ratios, and statistical significance reported.

**Overall Risk of Bias: Low**

- Strengths: Multi‑year temporal data, large sample sizes, WHO-standard methods, synergist assays, molecular genotyping, CI reporting.
- Weaknesses: None significant; comprehensive and robust study design.

**Ukpai & Ekedo, 2018 – Risk of Bias Assessment**

1. **Sample frame appropriate?** ✔ Yes — mosquitoes collected from Umudike, Abia State, a relevant endemic area.
2. **Sampling method appropriate?** ✔ Yes — larvae and pupae collected from natural breeding sites (gutters, pools, tyres, puddles), reared to adults under insectary conditions.
3. **Sample size adequate?** ✔ Yes — 20 females per replicate × 4 replicates = 80 per insecticide, plus controls. Meets WHO minimum thresholds.
4. **Species described in detail?** ✔ Yes — *Aedes aegypti* identified morphologically using Gillies & Coetzee keys. No molecular confirmation, but morphological description clear.
5. **Data collected directly?** ✔ Yes — WHO bioassays performed directly on reared adults.
6. **WHO methodology adhered?** ✔ Yes — WHO tube bioassays with discriminating doses (DDT 4%, bendiocarb 0.1%, primiphos-methyl 0.25%, deltamethrin 0.05%).
7. **Analysis appropriate?** ✔ Yes — mortality thresholds interpreted using WHO criteria; probit analysis used for KDT50/KDT90; ANOVA applied.
8. **Confounding factors addressed?** ✖ No — mosquito age (3–5 days old) reported, but insectary conditions (temperature, humidity) not fully detailed.
9. **Prevalence measure + CI reported?** ✖ No — mortality percentages reported, but confidence intervals not provided for bioassay outcomes.

**Overall Risk of Bias: Moderate**

- Strengths: WHO-standard bioassays, adequate sample size, clear species identification, probit analysis for knockdown times.
- Weaknesses: No CI reporting, limited control of confounders (environmental conditions not detailed).

**Ayorinde et al., 2015 – Risk of Bias Assessment**

1. **Sample frame appropriate?** ✔ Yes — mosquitoes collected from two farm sites (Badagry, Ikorodu) and one nonfarm site (Ebute Metta), representing both agricultural and urban settings in Lagos State.
2. **Sampling method appropriate?** ✔ Yes — larvae and pupae collected from tyres and containers, reared to adults under insectary conditions.
3. **Sample size adequate?** ✔ Yes — 80–100 mosquitoes per insecticide per site, with replicates, meeting WHO minimum thresholds.
4. **Species described in detail?** ✔ Yes — *Aedes aegypti* identified morphologically using Rueda (2004) pictorial keys. No molecular confirmation, but morphological identification clear.
5. **Data collected directly?** ✔ Yes — WHO tube bioassays performed directly on reared adults.
6. **WHO methodology adhered?** ✔ Yes — WHO tube bioassays with discriminating doses (DDT 4%, deltamethrin 0.05%, permethrin 0.75%). Mortality thresholds interpreted per WHO 1998 criteria.
7. **Analysis appropriate?** ✔ Yes — mortality percentages calculated, probit analysis for KDT50/KDT95, t‑tests applied. WHO thresholds correctly applied.
8. **Confounding factors addressed?** ✔ Yes — mosquito age (2–5 days old), insectary conditions (27–28 °C, 70–80% RH) controlled and reported.
9. **Prevalence measure + CI reported?** ✔ Yes — mortality percentages reported with confidence intervals for KDT values.

**Overall Risk of Bias: Low**

- Strengths: Multi‑site sampling, adequate sample size, WHO-standard bioassays, CI reporting, probit analysis.
- Weaknesses: Species identification morphological only, but acceptable given context.

**Ojianwuna et al., 2024 – Risk of Bias Assessment**

1. **Sample frame appropriate?** ✔ Yes — mosquitoes collected from multiple rural communities in Aniocha South LGA, Delta State (Ogwashi-uku, Nsukwa, Ejeme, Ubulu-uku). Relevant and representative.
2. **Sampling method appropriate?** ✔ Yes — larvae collected using WHO-standard dippers, reared to adults under insectary conditions, sex-sorted (focus on males).
3. **Sample size adequate?** ✔ Yes — 100 mosquitoes per insecticide per assay, with replicates, meeting WHO minimum thresholds.
4. **Species described in detail?** ✔ Yes — *Aedes aegypti* identified morphologically using Rueda keys; *Anopheles gambiae* identified using Coetzee keys. No molecular confirmation, but clear morphological description.
5. **Data collected directly?** ✔ Yes — WHO tube bioassays performed directly on reared adults; synergist assays with PBO included.
6. **WHO methodology adhered?** ✔ Yes — WHO bioassay protocols followed (DDT 4%, lambda‑cyhalothrin 0.05%), mortality thresholds interpreted per WHO criteria.
7. **Analysis appropriate?** ✔ Yes — mortality thresholds applied, probit analysis for KDT50/KDT95, ANOVA used, confidence intervals reported.
8. **Confounding factors addressed?** ✔ Yes — mosquito age (2 days old), insectary conditions controlled, feeding standardized.
9. **Prevalence measure + CI reported?** ✔ Yes — mortality percentages with 95% CI reported for knockdown times and resistance assays.

**Overall Risk of Bias: Low**

- Strengths: Multi‑site sampling, adequate sample size, WHO-standard bioassays, CI reporting, synergist assays, statistical analysis.
- Weaknesses: Species identification morphological only, but acceptable given context.

**Fagbohun et al., 2020 – Risk of Bias Assessment**

1. **Sample frame appropriate?** ✔ Yes — mosquitoes collected from four LGAs in Lagos State (Alimosho, Kosofe, Badagry, Ibeju-Lekki), representing both densely and sparsely populated areas.
2. **Sampling method appropriate?** ✔ Yes — larvae collected from peri-domestic habitats (water pots, tyre holes, abandoned wells), reared to adults under insectary conditions.
3. **Sample size adequate?** ✔ Yes — 100 mosquitoes per insecticide per site, with replicates, meeting WHO minimum thresholds.
4. **Species described in detail?** ✔ Yes — *Aedes aegypti* identified morphologically. No molecular confirmation, but morphological identification clear.
5. **Data collected directly?** ✔ Yes — WHO bioassays, PBO synergist assays, and biochemical enzyme assays performed directly on collected mosquitoes.
6. **WHO methodology adhered?** ✔ Yes — WHO tube bioassays with discriminating doses (DDT 4%, permethrin 0.75%, bendiocarb 0.1%), plus PBO synergist assays.
7. **Analysis appropriate?** ✔ Yes — mortality thresholds interpreted using WHO criteria; probit regression for KDT50/KDT95; chi‑square and regression analysis applied.
8. **Confounding factors addressed?** ✔ Yes — mosquito age (2–3 days old), insectary conditions (27 °C ± 2, RH 80% ± 10) controlled and reported.
9. **Prevalence measure + CI reported?** ✔ Yes — mortality percentages reported, confidence intervals provided for knockdown times and enzyme activity regressions.

**Overall Risk of Bias: Low**

- Strengths: Multi‑site sampling, adequate sample size, WHO-standard bioassays, CI reporting, synergist assays, biochemical assays.
- Weaknesses: Species identification morphological only, but acceptable given context.

**Fagbohun et al., 2021 – Risk of Bias Assessment**

1. **Sample frame appropriate?** ✔ Yes — mosquitoes collected from two peri‑domestic communities (Majidun and Oke‑Ota) in Ikorodu LGA, Lagos State. Relevant and representative.
2. **Sampling method appropriate?** ✔ Yes — larvae collected from breeding sites (tyres, containers, shallow wells), reared to adults under insectary conditions.
3. **Sample size adequate?** ✔ Yes — 80–100 mosquitoes per insecticide per site, with replicates, meeting WHO minimum thresholds.
4. **Species described in detail?** ✔ Yes — *Aedes aegypti* identified morphologically; molecular assays performed for kdr mutations (F1534C, S989P, V1016G).
5. **Data collected directly?** ✔ Yes — WHO bioassays performed directly on reared adults; PCR assays conducted for mutation detection.
6. **WHO methodology adhered?** ✔ Yes — WHO tube bioassays with discriminating doses (DDT 4%, permethrin 0.75%, deltamethrin 0.05%, lambda‑cyhalothrin 0.05%, bendiocarb 0.1%, malathion 5%).
7. **Analysis appropriate?** ✔ Yes — mortality thresholds applied, probit analysis for KDT50/KDT95, chi‑square tests, Hardy–Weinberg equilibrium analysis for Ace1R, PCR genotyping for kdr mutations.
8. **Confounding factors addressed?** ✔ Yes — mosquito age (2–5 days old), insectary conditions controlled, feeding standardized.
9. **Prevalence measure + CI reported?** ✔ Yes — mortality percentages reported, confidence intervals provided for knockdown times.

**Overall Risk of Bias: Low**

- Strengths: Multi‑site sampling, WHO-standard bioassays, molecular confirmation of resistance mechanisms, CI reporting, robust statistical analysis.
- Weaknesses: None significant; comprehensive design and reporting.
