# Supplementary material for: Systematic review and meta-analysis of insecticide resistance status and mechanisms in the arbovirus vector Aedes aegypti from Nigeria
Source: PLoS Negl Trop Dis. 2026 Jun 15;20(6):e0014421. doi: 10.1371/journal.pntd.0014421 (PMC13278583; doi:10.1371/journal.pntd.0014421)
Supplement: Attachment — Submitted filename: RESPONSE TO REVIEWRS_AM260426 (1).docx [file pntd.0014421.s010.docx]

**RESPONSE TO REVIEWERS'**	**COMMENTS**

**Dear Reviewers and Editorial Team,**

We sincerely thank the reviewers for their thorough evaluation of our manuscript and for the constructive comments and insightful suggestions provided. We appreciate the time and effort invested in helping us improve the quality, clarity, and scientific rigor of our work.

In response, we have carefully revised the manuscript and addressed each comment in detail. All changes have been clearly indicated, and we have provided point-by-point responses to ensure transparency and completeness. We believe that these revisions have significantly strengthened the manuscript and improved its overall impact.

We are grateful for the opportunity to revise our work and hope that the changes meet the expectations of the reviewers and the editorial team.

Reviewer #2 comment: “This review, which addresses insecticide resistance status and mechanisms in the arbovirus vector Aedes mosquitoes from Nigeria, provides a wealth of information on the status of resistance to synthetic insecticides in Nigeria. The methodology used is appropriate and allows for an analysis of differences in the susceptibility of Aedes aegypti across regions and insecticide types. However, some revisions are still needed to improve the quality of the document.”

Response: We thank the reviewer for their positive assessment of our study and for highlighting areas for improvement. In response, we have revised the Methods section to improve clarity and transparency:

- Objectives and hypothesis: We added an explicit statement of objectives and hypothesis at the start of the Methods section (page 6, lines 90-93).
- Study population: We clarified that the review focused on field-collected adult female Aedes aegypti mosquitoes from Nigeria, tested using standardized WHO bioassay protocols, to ensure relevance to arbovirus transmission risk (section 2.4.1).
- Statistical analyses: We expanded section 2.6 to emphasize why the chosen transformations, random-effects model, heterogeneity measures, and publication bias assessments are appropriate for prevalence data, thereby ensuring robust pooled estimates.
- Ethical/regulatory requirements: Section 2.7 confirms that ethical approval was verified for each included study and adherence to ethical guidelines was assessed.
- Supplementary materials: To enhance transparency, we referenced supplementary tables: “All studies identified through the search were tabulated (S1 Table), with reasons for exclusion provided. Data extraction was performed using a standardized form (S2 Table). Risk of bias assessments are summarized in S3 Table.” (Section 2.5.).

Reviewer #2 comment: “Results: put the meaning of DDT, PBO, S989P; and F1534C in the bottom of Table 1. Insecticide Susceptibility Profile: This section warrants the author presenting the stages of resistant Aedes mosquitoes; is it adult or larva? The authors mention Temephos and other insecticides. Furthermore, the methods used to assess vector resistance should be specified. The text presenting Table 2 should be written before Table 2. Also, as the references are not lot, I think author could add references in Table 2 not number of studies. Use portrait format to present Table 2. The quality of certain figures needs to be improved to make them more readable. Could you specify which kind of cytochrome P450 monooxygenases were detected in Nigeria?”

Response: We thank the reviewer for these constructive comments and have made the following revisions to strengthen the Results section:

- Table 1 footnotes: We added explanatory footnotes defining DDT (Dichlorodiphenyltrichloroethane), PBO (Piperonyl butoxide), F1534C (knockdown resistance mutation in the sodium channel gene: phenylalanine → cysteine at position 1534), and S989P (knockdown resistance mutation in the sodium channel gene: serine → proline at position 989).
- Stage clarification and methods: We specified that resistance assays were conducted on adult female Aedes aegypti, except for larval bioassays with temephos, which targeted the immature stage (line 216 page 16). We also cross-referenced the Methods section where WHO bioassay protocols are described, ensuring that the procedures used to assess vector resistance are clearly stated.
- Table 2 corrections: The descriptive text introducing Table 2 has been moved before the table. The table has been reformatted into portrait orientation, and the “Number of Studies (k)” column has been replaced with references to the specific studies contributing to each pooled estimate. We clarified in the narrative that pooled estimates are based on datasets, which may exceed the number of unique studies because multiple insecticides within a class were tested.
- Figures: We improved the resolution and labeling of Figures 2–8 to enhance readability.
- Cytochrome P450 specification: We clarified that metabolic resistance was linked to elevated activity of cytochrome P450 monooxygenases, as demonstrated by synergist assays with piperonyl butoxide (PBO). Specific isoforms were not consistently identified across Nigerian studies, and we have noted this limitation in the Results section.

Reviewer #2 comment: “Conclusion are good and limitations are clearly described. The discussion given by author will help for future researches.”

- Response: We thank the reviewer for their positive assessment of the conclusions and discussion. We are pleased that the conclusions were judged to be well-supported by the data and that the limitations were clearly described. We have ensured that the conclusions highlight the public health relevance of widespread insecticide resistance in Aedes aegypti across Nigeria, emphasizing the urgent need for adaptive vector control strategies and expanded surveillance. The discussion also outlines how these findings can guide future research, particularly in integrating genomic tools and strengthening monitoring systems. No further changes were required in this section, as the reviewer found it satisfactory.

Reviewer #2 comment: “This review, which addresses insecticide resistance status and mechanisms in the arbovirus vector Aedes mosquitoes from Nigeria, provides a wealth of information on the status of resistance to synthetic insecticides in Nigeria. The methodology used is appropriate and allows for an analysis of differences in the susceptibility of Aedes aegypti across regions and insecticide types. However, some revisions are still needed to improve the quality of the document. Abstract: abbreviate the name Aedes after writing the entirely first time. Background: line 84 abbreviate the name Aedes. What is being done in Nigeria to combat the resistance phenomenon? Are there any strategies, or despite the resistance, is nothing being done to counter its effects? If new strategies are being used, please describe them briefly in the introduction. Line 66: ‘Effective vector control relies heavily on insecticide-based interventions’ Provide more details about this sentence and cite a source.”

Response: We thank the reviewer for these constructive editorial suggestions and have made the following modifications to improve clarity and contextual relevance:

- Abstract and Background: We abbreviated Aedes aegypti and Aedes albopictus to Ae. aegypti and Ae. albopictus after their first full mention in both the Abstract and Background sections.
- Introduction – strategies in Nigeria: We added a brief description of current strategies being implemented in Nigeria to combat insecticide resistance. Specifically, we noted that integrated vector management (IVM) approaches are being piloted, including larval source management, environmental sanitation, and the use of synergist-based formulations (pyrethroids combined with piperonyl butoxide [PBO]) to partially restore susceptibility (line 62-68). We also highlighted that surveillance programs are expanding in some states, though coverage remains limited. These additions were supported with references to the National Malaria Elimination Programme (NMEP, 2021), the National Malaria Strategic Plan (2021–2025), and recent Nigerian entomological studies.
- We expanded the sentence on insecticide-based interventions to specify that these include indoor residual spraying, space spraying, and larval control measures, and we cited WHO (2020) and Nigeria’s National Malaria Elimination Programme guidelines to provide authoritative references.

Reviewer #1 comment: *“Comments on the Manuscript PNTD-D-25-02248 entitled ‘Systematic review and meta-analysis of insecticide resistance status and mechanisms in the arbovirus vector Aedes mosquitoes from Nigeria.’ This study is a systematic review and meta-analysis that aims to assess the insecticides resistance status and involved mechanisms in Aedes populations in Nigeria. The topic is of clear public health interest but these specific points need to be clarified:

- The manuscript should focus specifically on Aedes aegypti, and the title revised accordingly
- The genus Aedes should be abbreviated after the first appearance in both the abstract and the main text, except when it occurs at the beginning of a sentence.
- A map showing the study locations would be helpful to visualize the geographic distribution of the reviewed studies.
- Figure legends are missing and should be added
- Knockdown resistance (kdr) should be defined at its first occurrence, and the abbreviation ‘kdr’ used thereafter.
- L90: please correct the typo.”*

Response: We thank the reviewer for their constructive comments and have made the following revisions to strengthen the manuscript:

- Title revision: We revised the title to focus specifically on Aedes aegypti, as this was the primary species analyzed in the systematic review and meta-analysis.
- Abbreviation of genus: We ensured that Aedes aegypti and Aedes albopictus are written in full at first mention in both the Abstract and main text, and abbreviated to Ae. aegypti and Ae. albopictus thereafter, except when appearing at the beginning of a sentence.
- Map addition: We added a map figure showing the geographic distribution of the reviewed studies across Nigeria, which enhances visualization of regional coverage and variability.
- Figure legends: We added clear and descriptive legends to all figures to improve readability and interpretation.
- Knockdown resistance definition: We defined knockdown resistance (kdr) at its first occurrence in the text (line 75-78) as “mutations in the voltage-sensitive sodium channel gene that confer target-site insensitivity,” and thereafter consistently used the abbreviation “kdr.”
- Typographical correction: We corrected the typo at line 90.
