# Supplementary material for: Systematic review and meta-analysis of insecticide resistance status and mechanisms in the arbovirus vector Aedes aegypti from Nigeria
Source: PLoS Negl Trop Dis. 2026 Jun 15;20(6):e0014421. doi: 10.1371/journal.pntd.0014421 (PMC13278583; doi:10.1371/journal.pntd.0014421)
Supplement: Peer review history

# Peer review: Decision Letter 0

**DOI:** 10.1371/journal.pntd.0014421.r001

**Authors:** Vincent Corbel, Adly Abd-Alla

16 Apr 2026

PNTD-D-25-02248 Systematic review and meta-analysis of insecticide resistance status and mechanisms in the arbovirus vector aedes mosquitoes from Nigeria PLOS Neglected Tropical Diseases Dear Dr. Aisha, Thank you for submitting your manuscript to PLOS Neglected Tropical Diseases. After careful consideration, we feel that it has merit but does not fully meet PLOS Neglected Tropical Diseases's publication criteria as it currently stands. Therefore, we invite you to submit a revised version of the manuscript that addresses the points raised during the review process. Please submit your revised manuscript by 14 days. If you will need more time than this to complete your revisions, please reply to this message or contact the journal office at plosntds@plos.org. When you're ready to submit your revision, log on to https://www.editorialmanager.com/pntd/ and select the 'Submissions Needing Revision' folder to locate your manuscript file. Please include the following items when submitting your revised manuscript: * A letter that responds to each point raised by the editor and reviewer(s). You should upload this letter as a separate file labeled 'Response to Reviewers'. This file does not need to include responses to any formatting updates and technical items listed in the 'Journal Requirements' section below.* A marked-up copy of your manuscript that highlights changes made to the original version. You should upload this as a separate file labeled 'Revised Manuscript with Track Changes'.* An unmarked version of your revised paper without tracked changes. You should upload this as a separate file labeled 'Manuscript'. If you would like to make changes to your financial disclosure, competing interests statement, or data availability statement, please make these updates within the submission form at the time of resubmission. Guidelines for resubmitting your figure files are available below the reviewer comments at the end of this letter.

We look forward to receiving your revised manuscript.

Kind regards, Vincent CorbelAcademic EditorPLOS Neglected Tropical Diseases Adly Abd-AllaSection EditorPLOS Neglected Tropical Diseases

Shaden Kamhawi

co-Editor-in-Chief

PLOS Neglected Tropical Diseases

orcid.org/0000-0003-4304-636XX

Paul Brindley

co-Editor-in-Chief

PLOS Neglected Tropical Diseases

orcid.org/0000-0003-1765-0002

**Journal Requirements:**

1) Please ensure that the CRediT author contributions listed for every co-author are completed accurately and in full.

At this stage, the following Authors/Authors require contributions: Aisha Kabir Mohammed, Maryam Abdulkadir Dangambo, Aliyu Muhammad, and Adamu Jibrin Alhassan. Please ensure that the full contributions of each author are acknowledged in the "Add/Edit/Remove Authors" section of our submission form.

The list of CRediT author contributions may be found here: https://journals.plos.org/plosntds/s/authorship#loc-author-contributions

2) As required by our policy on Data Availability, please ensure your manuscript or supplementary information includes the following:

- A numbered table of all studies identified in the literature search, including those that were excluded from the analyses.

- For every excluded study, the table should list the reason(s) for exclusion.

- If any of the included studies are unpublished, include a link (URL) to the primary source or detailed information about how the content can be accessed.

- A table of all data extracted from the primary research sources for the systematic review and/or meta-analysis. The table must include the following information for each study:

- Name of data extractors and date of data extraction

- Confirmation that the study was eligible to be included in the review.

- All data extracted from each study for the reported systematic review and/or meta-analysis that would be needed to replicate your analyses.

- If data or supporting information were obtained from another source (e.g. correspondence with the author of the original research article), please provide the source of data and dates on which the data/information were obtained by your research group.

- If applicable for your analysis, a table showing the completed risk of bias and quality/certainty assessments for each study or outcome.  Please ensure this is provided for each domain or parameter assessed. For example, if you used the Cochrane risk-of-bias tool for randomized trials, provide answers to each of the signalling questions for each study. If you used GRADE to assess certainty of evidence, provide judgements about each of the quality of evidence factor. This should be provided for each outcome.

- An explanation of how missing data were handled.

This information can be included in the main text, supplementary information, or relevant data repository.

Note: If the reviewer comments include a recommendation to cite specific previously published works, please review and evaluate these publications to determine whether they are relevant and should be cited. There is no requirement to cite these works unless the editor has indicated otherwise.

**Reviewers' comments:** Reviewer's Responses to Questions

**Key Review Criteria Required for Acceptance?**

As you describe the new analyses required for acceptance, please consider the following:

**Methods**

-Are the objectives of the study clearly articulated with a clear testable hypothesis stated?

-Is the study design appropriate to address the stated objectives?

-Is the population clearly described and appropriate for the hypothesis being tested?

-Is the sample size sufficient to ensure adequate power to address the hypothesis being tested?

-Were correct statistical analysis used to support conclusions?

-Are there concerns about ethical or regulatory requirements being met?

Reviewer #1: (No Response)

Reviewer #2: This review, which addresses insecticide resistance status and mechanisms in the arbovirus vector aedes mosquitoes from Nigeria., provides a wealth of information on the status of resistance to synthetic insecticides in Nigeria. The methodology used is appropriate and allows for an analysis of differences in the susceptibility of Aedes aegypti across regions and insecticide types. However, some revisions are still needed to improve the quality of the document.

**********

**Results**

-Does the analysis presented match the analysis plan?

-Are the results clearly and completely presented?

-Are the figures (Tables, Images) of sufficient quality for clarity?

Reviewer #1: (No Response)

Reviewer #2: This review, which addresses insecticide resistance status and mechanisms in the arbovirus vector aedes mosquitoes from Nigeria., provides a wealth of information on the status of resistance to synthetic insecticides in Nigeria. The methodology used is appropriate and allows for an analysis of differences in the susceptibility of Aedes aegypti across regions and insecticide types. However, some revisions are still needed to improve the quality of the document.

Results: put the meaning of DDT, PBO, S989P; and F1534C in the bottom of table 1

Insecticide Susceptibility Profile: This section warrants the author presenting the stages of resistant Aedes mosquitoes; is it adult or larva? The authors mention Temephos and other insecticides. Furthermore, the methods used to assess vector resistance should be specified.

The text presenting table 2 should be write before table2. Also, as the reference are not lot, I think author could add references in table 2 not number of studies.

Use portrait format to present Table 2

The quality of certain figures needs to be improved to make them more readable.

Could you specify which kind of cytochrome P450 monooxygenases were detected in Nigeria?

**********

**Conclusions**

-Are the conclusions supported by the data presented?

-Are the limitations of analysis clearly described?

-Do the authors discuss how these data can be helpful to advance our understanding of the topic under study?

-Is public health relevance addressed?

Reviewer #1: (No Response)

Reviewer #2: conclusion are good and limitations are clearly described. the discussion given by author will help for future researches

**********

**Editorial and Data Presentation Modifications?**

Use this section for editorial suggestions as well as relatively minor modifications of existing data that would enhance clarity. If the only modifications needed are minor and/or editorial, you may wish to recommend “Minor Revision” or “Accept”.

Reviewer #1: (No Response)

Reviewer #2: This review, which addresses insecticide resistance status and mechanisms in the arbovirus vector aedes mosquitoes from Nigeria., provides a wealth of information on the status of resistance to synthetic insecticides in Nigeria. The methodology used is appropriate and allows for an analysis of differences in the susceptibility of Aedes aegypti across regions and insecticide types. However, some revisions are still needed to improve the quality of the document.

Abtract: abbreviate the name Aedes after writing the entirely first time

Background: line 84 abbreviate the name Aedes

What is being done in Nigeria to combat the resistance phenomenon? Are there any strategies, or despite the resistance, is nothing being done to counter its effects? If new strategies are being used, please describe them briefly in the introduction.

Line 66:” Effective vector control relies heavily on insecticide-based interventions” Provide more details about this sentence and cite a source.

**********

**Summary and General Comments**

Use this section to provide overall comments, discuss strengths/weaknesses of the study, novelty, significance, general execution and scholarship. You may also include additional comments for the author, including concerns about dual publication, research ethics, or publication ethics. If requesting major revision, please articulate the new experiments that are needed.

Reviewer #1: Comments on the Manuscript PNTD-D-25-02248 entitled “Systematic review and meta-analysis of insecticide resistance status and mechanisms in the arbovirus vector aedes mosquitoes from Nigeria”

This study is a systematic review and meta-analysis that aims to assess the insecticides resistance status and involved mechanisms in Aedes populations in Nigeria. The topic is of clear public health interest but these specific points need to be clarified:

- The manuscript should focus specifically on Aedes aegypti, and the title revised accordingly

- The genus Aedes should be abbreviated after the first appearance in both the abstract and the main text, except when it occurs at the beginning of a sentence.

- A map showing the study locations would be helpful to visualize the geographic distribution of the reviewed studies.

- Figure legends are missing and should be added

- knockdown resistance (kdr) should be defined at its first occurrence, and the abbreviation “kdr” used thereafter.

- L90: please correct the typo

Reviewer #2: (No Response)

**********

PLOS authors have the option to publish the peer review history of their article (what does this mean?). If published, this will include your full peer review and any attached files.

If you choose “no”, your identity will remain anonymous but your review may still be made public.

**Do you want your identity to be public for this peer review?** For information about this choice, including consent withdrawal, please see our Privacy Policy.

Reviewer #1: No

Reviewer #2: **Yes:**Wendgida Dimitri WANGRAWA

[NOTE: If reviewer comments were submitted as an attachment file, they will be attached to this email and accessible via the submission site. Please log into your account, locate the manuscript record, and check for the action link "View Attachments". If this link does not appear, there are no attachment files.]**Figure resubmission:** While revising your submission, we strongly recommend that you use PLOS’s NAAS tool (https://ngplosjournals.pagemajik.ai/artanalysis) to test your figure files. NAAS can convert your figure files to the TIFF file type and meet basic requirements (such as print size, resolution), or provide you with a report on issues that do not meet our requirements and that NAAS cannot fix.

After uploading your figures to PLOS’s NAAS tool - https://ngplosjournals.pagemajik.ai/artanalysis, NAAS will process the files provided and display the results in the "Uploaded Files" section of the page as the processing is complete. If the uploaded figures meet our requirements (or NAAS is able to fix the files to meet our requirements), the figure will be marked as "fixed" above. If NAAS is unable to fix the files, a red "failed" label will appear above. When NAAS has confirmed that the figure files meet our requirements, please download the file via the download option, and include these NAAS processed figure files when submitting your revised manuscript.**Reproducibility:** To enhance the reproducibility of your results, we recommend that authors of applicable studies deposit laboratory protocols in protocols.io, where a protocol can be assigned its own identifier (DOI) such that it can be cited independently in the future. Additionally, PLOS ONE offers an option to publish peer-reviewed clinical study protocols. Read more information on sharing protocols at https://plos.org/protocols?utm_medium=editorial-email&utm_source=authorletters&utm_campaign=protocols

# Author comment: Author response to Decision Letter 1

**DOI:** 10.1371/journal.pntd.0014421.r002

28 Apr 2026

Attachment Submitted filename: RESPONSE TO REVIEWRS_AM260426 (1).docx

# Editor report: Decision Letter 1

**DOI:** 10.1371/journal.pntd.0014421.r003

**Authors:** Vincent Corbel, Adly Abd-Alla, Vincent Corbel, Audrey Lenhart

28 May 2026

Dear Mrs Mohammed,

We are pleased to inform you that your manuscript 'Systematic review and meta-analysis of insecticide resistance status and mechanisms in the arbovirus vector aedes aegypti from Nigeria' has been provisionally accepted for publication in PLOS Neglected Tropical Diseases.

Before your manuscript can be formally accepted you will need to complete some formatting changes, which you will receive in a follow up email. A member of our team will be in touch with a set of requests.

Please note that your manuscript will not be scheduled for publication until you have made the required changes, so a swift response is appreciated.

IMPORTANT: The editorial review process is now complete. PLOS will only permit corrections to spelling, formatting or significant scientific errors from this point onwards. Requests for major changes, or any which affect the scientific understanding of your work, will cause delays to the publication date of your manuscript.

Should you, your institution's press office or the journal office choose to press release your paper, you will automatically be opted out of early publication. We ask that you notify us now if you or your institution is planning to press release the article. All press must be co-ordinated with PLOS.

Thank you again for supporting Open Access publishing; we are looking forward to publishing your work in PLOS Neglected Tropical Diseases.

Best regards,

Vincent Corbel

Academic Editor

PLOS Neglected Tropical Diseases

Adly Abd-Alla

Section Editor

PLOS Neglected Tropical Diseases

Shaden Kamhawi

co-Editor-in-Chief

PLOS Neglected Tropical Diseases

orcid.org/0000-0003-4304-636XX

Paul Brindley

co-Editor-in-Chief

PLOS Neglected Tropical Diseases

orcid.org/0000-0003-1765-0002

***********************************************************

Dear Dr Mohammed,

Many thanks for revising the manuscript and providing a cover letter explaining the modifications made to the article.

After reviewing your revised manuscript, we are pleased to inform you that it meets the standards for publication in Plos Neglected Tropical Diseases

Again, thank you for collaborating with us.

With best wishes,

Vincent Corbel

Academic Editor

# Editor report: Acceptance letter

**DOI:** 10.1371/journal.pntd.0014421.r004

**Authors:** Vincent Corbel, Adly Abd-Alla, Vincent Corbel, Audrey Lenhart

Dear Mrs Mohammed,

We are delighted to inform you that your manuscript, "Systematic review and meta-analysis of insecticide resistance status and mechanisms in the arbovirus vector Aedes aegypti from Nigeria," has been formally accepted for publication in PLOS Neglected Tropical Diseases.

We have now passed your article onto the PLOS Production Department who will complete the rest of the publication process. All authors will receive a confirmation email upon publication.

The corresponding author will soon be receiving a typeset proof for review, to ensure errors have not been introduced during production. Please review the PDF proof of your manuscript carefully, as this is the last chance to correct any scientific or type-setting errors. Please note that major changes, or those which affect the scientific understanding of the work, will likely cause delays to the publication date of your manuscript. Note: Proofs for Front Matter articles (Editorial, Viewpoint, Symposium, Review, etc...) are generated on a different schedule and may not be made available as quickly.

Soon after your final files are uploaded, the early version of your manuscript will be published online unless you opted out of this process. The date of the early version will be your article's publication date. The final article will be published to the same URL, and all versions of the paper will be accessible to readers.

For Research Articles, you will receive an invoice from PLOS for your publication fee after your manuscript has reached the completed accept phase. If you receive an email requesting payment before acceptance or for any other service, this may be a phishing scheme. Learn how to identify phishing emails and protect your accounts at https://explore.plos.org/phishing.

Thank you again for supporting open-access publishing; we are looking forward to publishing your work in PLOS Neglected Tropical Diseases.

Best regards,

Shaden Kamhawi

co-Editor-in-Chief

PLOS Neglected Tropical Diseases

Paul Brindley

co-Editor-in-Chief

PLOS Neglected Tropical Diseases
